# Cryo-EM structure of locked spike glycoprotein from bat SARS-like coronavirus WIV1, molecular dynamics and biophysics across host range

**DOI:** 10.1073/pnas.2516874123

**Published:** 2026-02-18

**Authors:** Chuan Liu, Jingjing Zheng, Yuhan Wang, Florian Beck, István Nagy, Stefan Bohn, Jürgen M. Plitzko, Wolfgang Baumeister, Xiaoxiao Zhang, Liping Sun, Luca Zinzula

**Affiliations:** ^a^iHuman Institute ShanghaiTech University, Shanghai 201210, China; ^b^Max Planck Institute of Biochemistry, Emeritus Group Molecular Structural Biology, Martinsried 82152, Germany; ^c^School of Life Sciences and Technology, ShanghaiTech University, Shanghai 201210, China; ^d^Max Planck Institute of Biochemistry, Research Group CryoEM Technology, Martinsried 82152, Germany; ^e^Center of Research and Development, Eszterházy Károly Catholic University, Eger 3300, Hungary; ^f^CryoEM Platform and Institute of Structural Biology, Helmholtz Munich, Oberschleissheim 85764, Germany

**Keywords:** bat SARS-like coronavirus, cryo-EM, molecular dynamics, spike glycoprotein, WIV1

## Abstract

As made evident by severe acute respiratory syndrome coronavirus 1 (SARS-CoV-1) and SARS-CoV-2 pandemics, the possibility of a SARS-like coronavirus (SL-CoV) emerging again in humankind after zoonotic spillover represents a significant global health threat. Given the role of spike (S) glycoprotein in mediating SL-CoV cell entry and cross-species transmission, there remains urgent need of structural information on SL-CoV S to guide therapeutic countermeasure development. Among SL-CoVs, bat-derived WIV1 is capable of using as receptor the angiotensin converting enzyme 2 (ACE2) from a variety of mammals, thereby representing a prototype model for studying SL-CoV precursors to future pandemics. We present a WIV1 S cryo-EM structure in prefusion state which reveals molecular signatures reminiscent of the tightly-packed locked-1 conformation described for SARS-CoV-1 and SARS-CoV-2. To decipher the molecular basis for bat SL-CoV WIV1 host range tropism, we performed molecular dynamics (MD) simulations of WIV1 S-ACE2 interaction across reservoir bat, potentially intermediate hosts civet, raccoon dog and pangolin, and accidental human hosts. We found that, in all interactions, upon complex formation with ACE2, the linoleic acid responsible for locking the S receptor binding domain (RBD) dynamically persists in its binding pocket, however repositioning to potentially unlock the system. Complex formation between WIV1 S-RBD and ACE2 from different hosts was characterized in vitro by mass photometry and microscale thermophoresis, revealing that interaction is stronger with ACE2 from bat and human than other hosts, within the latter stronger for the Thr92Ile polymorphism correlated to higher SARS-CoV-2 infection susceptibility. These findings provide critical insights with crucial implications for pandemic preparedness.

## Significance

Understanding how SARS-like coronaviruses (SL-CoVs) spill over into human population is critical for anticipating and preventing future pandemics. WIV1 is a concerning SL-CoV, given its capability to infect multiple mammals. The herein presented WIV1 spike (S) glycoprotein cryo-EM structure adopts the linoleic acid (LA)-bound, tightly-packed conformation previously described in SARS-CoV-1 and SARS-CoV-2 as “locked.” Molecular dynamics shows that LA repositioning tunes the way WIV1 S receptor binding domain (RBD) engages to angiotensin converting enzyme 2 (ACE2) receptor from reservoir, potential intermediate and accidental human hosts. Biophysical characterization illustrates a binding affinity gradient across hosts, revealing molecular features underlying WIV1 cross-species transmission. This work provides structural and mechanistic insights into SL-CoV host range, identifying key interactions to inform surveillance and therapeutic strategies.

The zoonotic spillover into the human population of novel severe acute respiratory syndrome (SARS)-like coronaviruses (SL-CoVs) represents a persisting threat to global health, made tangible by two pandemic events occurred during the past decades such as the 2002-03 by SARS-CoV-1 and the recent COVID-19 (Covid-19) by SARS-CoV-2 (1). SL-CoVs are enveloped, nonsegmented, positive-sense single-stranded RNA viruses that phylogenetically belong to four clades within the *Sarbecovirus* subgenus of the *Betacoronavirus* genus in the *Coronavirinae* subfamily (family *Coronaviridae*, order *Nidovirales*) (2). SL-CoVs have in chiropterans their natural reservoirs; nevertheless, some of them can also infect other mammalians and potentially jump to humans directly from the bat or via intermediate hosts (3). Such broad host range tropism derives from the properties of their spike (S) glycoprotein, which mediates virus attachment and subsequent fusion to the host cell membrane by exploiting an array of angiotensin-converting enzyme 2 (ACE2) orthologs as receptors (2, 4). Therefore, given their critical role in determining coronavirus cross-species transmission and evolution, structural characterization of S glycoproteins across SL-CoVs is of utmost importance. In the wake of the Covid-19 pandemic, tremendous efforts have been put toward understanding the mechanistic details of SARS-CoV-2 entry in host cells, which led to dozens of structures of its S glycoprotein deposited in the Protein Data Bank (PDB), determined either as isolated homotrimers or in complex with the receptor ACE2 from human and other animal species, as well as bound to monoclonal antibodies (mAbs) (5). Overall, homotrimeric S glycoprotein structures from SARS-CoV-1 and SARS-CoV-2 have been determined in two functional states, namely the mushroom-like prefusion state—either in the conformation denoted as “closed,” with three copies of the receptor binding domain (RBD) all in “down” position, or in those defined as “open,” with one or more RBDs in “up” position (6–9)—and the hairpin-like postfusion state (10–13). Noteworthy, both states were shown to coexist on the surface of intact virions (14–19). Within the prefusion state, another metastable conformation of the S glycoprotein was identified and designated as “locked” in regard to the more rigid and tightly packed organization of its homotrimer, whose RBDs are all kept in “down” position and restrained in their movements by the presence of lipid ligands and interdomain interactions (20, 21). At the level of the single S protomer, two subtypes of this conformation were further characterized and termed as “locked-1” and “locked-2,” respectively. In turn, this led to the identification of various combinations in the context of the S homotrimer, that could be categorized as either symmetric, i.e., with three protomers all in locked-1 or locked-2 conformation, or asymmetric, i.e., with two protomers in locked-1 (locked-112) or in locked-2 (locked-122) conformation, respectively (22, 23). Besides, in addition to SARS-CoV-1 and SARS-CoV-2, light was shed on the structure of S glycoproteins from various SL-CoVs, including bat SL-CoVs RaTG13 (24, 25), RsSHC014 (26), BANAL-20-236 (PDB ID: 8I3W) and BANAL-20-52 (PDB ID: 8HXJ), pangolin SL-CoVs from the Chinese provinces Guangxi (PCoV-GX) (25) and Guangdong (PCoV-GD) (27), and civet SL-CoVs 007 and SZ3 (28). Moreover, closely related to those SL-CoVs—and particularly to SARS-CoV-1—is also WIV1, which was isolated in 2013 from samples of Chinese rufous horseshoe bat (*Rhinolophus sinicus*) collected in the Yunnan province, China, and is considered one of the most concerning SL-CoVs (29, 30). In fact, bat SL-CoV WIV1 can efficiently use ACE2 from human and several wildlife species, which makes it poised for emergence (31, 32). Until recently, structural knowledge on the S glycoprotein from bat SL-CoV WIV1 was limited, with the only available information coming from a crystallographic structure of its RBD complexed to a human antigen-binding fragment (Fab) mAb (33). Here, we present the structure of bat SL-CoV WIV1 S glycoprotein determined by cryogenic electron microscopy (cryo-EM) single particle analysis (SPA), which we captured in a tightly packed conformation that shows features similar to locked-1 SARS-CoV-1 and SARS-CoV-2 S structures, and discuss its differences with respect to orthologs from closely related SL-CoVs, as well as its uniqueness as compared to WIV1 S structures that were published during the preparation of this manuscript (26, 28). Moreover, by using as experimental model the herein determined WIV1 S RBD structure, we present an in-depth computational analysis of the molecular dynamics (MD) of the complex formed by WIV1 S RBD with ACE2 receptors from the natural reservoir horseshoe bat, the human accidental host and a panel of other mammalian species that may serve as intermediate hosts. Furthermore, in silico results were validated through biophysical characterization of WIV1 S RBD-ACE2 interaction, confirming the existence of a gradient in binding affinity toward receptors across the host tropism range.

## Results

### Overall Characteristics of Bat SL-CoV WIV1 S Structure.

**Fig. 1.**
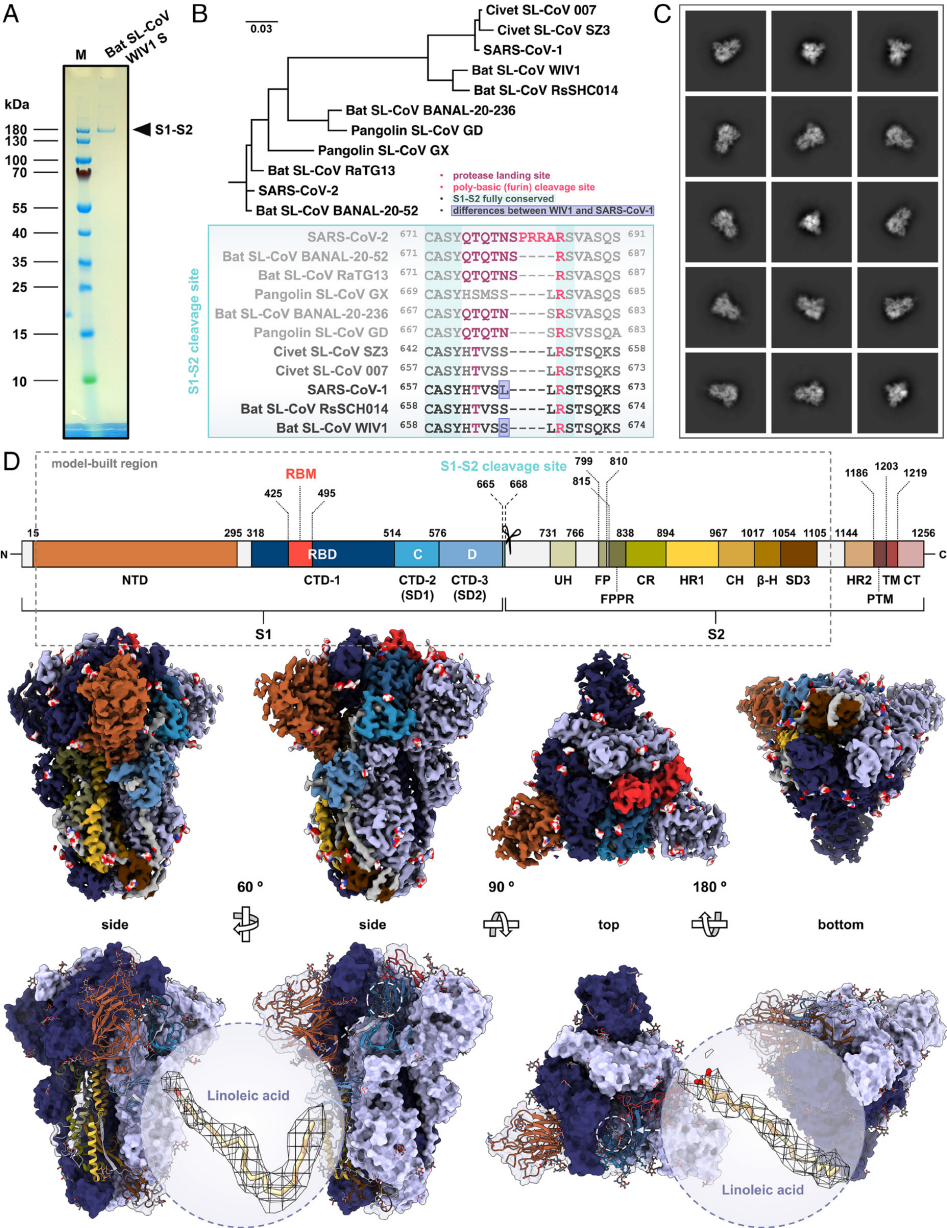
Structure of bat SL-CoV WIV1 S in locked-1-like conformation. (*A*) SDS-PAGE analysis of purified recombinant WIV1 S ectodomain (aa 20-1194), showing one band of uncleaved S1-S2 polypeptide (M, molecular weight marker). (*B*) Molecular evolutionary analysis of SL-CoV S proteins based on a maximum-likelihood phylogenetic tree (*Upper*) of the aa reference sequences from WIV1, SARS-CoV-1, SARS-CoV-2 and representative closely related SL-CoVs, and multiple sequence alignment of their S1-S2 cleavage site; fully conserved residues are highlighted with sea-serpent cyan background; residues differing between WIV1 and SARS-CoV-1 are boxed in slate blue; residues corresponding to the protease-landing and furin-cleavage sites are colored in amaranth deep purple and radical red, respectively. (*C*) representative 2D class averages of WIV1 S cryo-EM SPA showing random orientations of S homotrimers in prefusion state. (*D*) Structural organization (*Upper*) of WIV1 S monomer, and four-orientation rendering of cryo-EM density map (*Middle*) and corresponding atomic model (*Lower*) of its homotrimeric structure; residue positions of domain boundaries in the S monomer are indicated, with domains colored in orange and different shades of blue for the S1, and in different shades of green, yellow, and brown for the S2 subunits, respectively (see *SI Appendix*, Fig. S3 for domain name abbreviations and color details); in the EM-density map, two monomers are colored in deep koamaru and ceil violet, respectively, whereas a third one is colored following the domain structural organization; glycans are represented as spheres and colored by heteroatoms; in the atomic model, the former two monomers are shown as isosurface representation, whereas the latter one is shown as ribbon representation with RBD-bound LA ligand highlighted by dashed circles, also shown in zoomed *Insets* as stick representation fitted into the EM density map mesh.

Recombinant bat SL-CoV WIV1 S ectodomain was expressed in *Spodoptera frugiperda* (Sf) 9 insect cells as secreted protein, purified at homogeneity by affinity chromatography (AC) and size-exclusion chromatography (SEC), and plunge-frozen in amorphous vitreous ice for structure determination by cryo-EM SPA. Sodium dodecyl sulfate–polyacrylamide gel electrophoresis (SDS-PAGE) analysis showed that purified bat SL-CoV WIV1 S recombinant construct migrates as a single protein band of ∼180 kDa apparent molecular weight (MW), consistent with a heavy glycosylation of its monomer, that would have an expected theoretical MW of 134 kDa otherwise (Fig. 1*A*). Also, such one band-only migration profile agrees with the fact that, like its orthologs from closely related SARS-CoV-1 and other SL-CoVs, WIV1 S does not bear the polybasic motif recognized by furin protease (Fig. 1*B*), and therefore the S1-S2 cleavage site at amino acid (aa) positions 665-668 is not processed to split the protein into the S1 and S2 subunits during its cellular biogenesis (34, 35). Classification of two-dimensional (2D) averages of particle projections revealed that purified WIV1 S exists in solution as homogeneous population of homotrimeric proteins in prefusion state (Fig. 1*C*), whereas three-dimensional (3D) reconstruction led to a characteristic mushroom-like density map of 3.38 Å average final resolution (Fig. 1*D* and *SI Appendix*, Fig. S1). Atomic modeling of the homotrimeric S accurately assigned to the density map secondary structures of the glycoprotein (Fig. 1*D* and *SI Appendix*, Figs. S1 and S2), defining its topology and boundaries of 12 domains within the region that comprises residues 20-1120 of bat SL-CoV WIV1 S (Fig. 1*D* and *SI Appendix*, Fig. S3). Moreover, alignment of the corresponding aa sequence with that of S glycoproteins from closely related SL-CoVs whose structures have been already determined, showed that a high degree of conservation exists throughout the S2 subunit (modeled aa 668-1120), whereas the highest variability is present within the S1 subunit, especially in the N-terminal domain (NTD, modeled aa 20-295) and the receptor binding motif (RBM, aa 425-495) of the RBD (aa 318-514) (*SI Appendix*, Fig. S4). In addition, from the cryo-EM density map was possible to define the location of eighty-seven N-linked glycans per S trimer, including one α-D-mannose (MAN), two β-D-mannose (BMA) and 26 N-acetyl-β-D-glucosamine (NAG) molecules per monomeric chain, which in the atomic model branch out from seventeen glycosylation sites in each S monomer (Fig. 1*D* and *SI Appendix*, Figs. S2 and S5). Of note, these glycans differ by number, molecule type, or spatial orientation at given glycosylated residue position, with respect to those found in two WIV1 S cryo-EM structures recently described (*SI Appendix*, Fig. S2) (26, 28). With the caveat that orthogonal confirmation by mass spectrometry was not performed and that peculiarities of glycans may be also attributable to the different expression system adopted in this work compared to previous ones, as well as to the difference in resolution between the density maps, the accuracy of our cryo-EM density map allowed us to unequivocally detect glycans with 72.2% and 74.1% identity to those present in the WIV1 S expressed in Chinese hamster (PDB ID: 8TC0) and human (PDB ID: 8WQ0) cells, respectively (*SI Appendix*, Figs. S2 and S5). Furthermore, each RBD in our WIV1 S homotrimer accommodates one linoleic acid (LA) molecule in the so-called free fatty acid binding pocket (FFABP), where its tail is lined by a cluster of hydrophobic residues and its head is anchored to polar ones (Fig. 1*D* and *SI Appendix*, Fig. S2). The presence of LA in the FFABP was first detected in SARS-CoV-2 S and correlated to the stabilization of locked conformations, thereby preventing the transition of RBDs from “down” to “up” position that is required for its binding to ACE2 (21). Subsequently, LA-bound FFABPs were also reported for SARS-CoV-1 and other SL-CoVs, this structural feature being further recognized as distinctive trait shared by all highly pathogenic betacoronaviruses, and also hypothesized to be an exploitable antiviral target (36, 37). We then performed structural alignment of our bat SL-CoV WIV1 S to a panel of representative ortholog structures selected from the PDB for bearing the LA-bound FFABP and calculated the alpha-carbon (C-α) backbone RMSD values for the whole S trimer, the S1 NTD, the RBD or C-terminal domain 1 (CTD-1), the CTD-2 or subdomain 1 (SD1) or C-domain (aa 515-576), the CTD-3 or SD2 or D-domain (aa 577-664), and the S2 subunit (*SI Appendix*, Fig. S6). Results showed that our homotrimeric structure is similar to previously determined LA-bound WIV1 S (PDB ID: 8TC0), even though with relatively high RMSD values (1.338 Å) as compared to structures in the panel from other SL-CoVs. In particular, either for the entire S trimeric ectodomain (RMSD 0.716 Å), as well as for the isolated monomeric S1 NTD (0.618 Å), CTD-2 (0.318 Å), and S2 (0.602 Å), the highest structural similarity of bat SL-CoV WIV1 S is with SARS-CoV-1 orthologs (PDB IDs: 8H14 and 8H0X, respectively), whereas for the CTD-1 (0.456 Å) and CTD-3 (0.439 Å) is with civet SL-CoV 007 (PDB ID: 8TC1) and SARS-CoV-2 (PDB ID: 6ZB5) S structures, respectively (*SI Appendix*, Fig. S6). Furthermore, among the SARS-CoV-2 representatives, the one that best aligns to bat SL-CoV WIV1 S (PDB ID: 7OD3) shows an RMSD value of 1.231 Å. Noteworthy, among the most similar ones and better structurally aligned to WIV1 S, top-ranked SARS-CoV-1 (PDB ID: 8H14) and SARS-CoV-2 (PDB ID: 7OD3) orthologs are both reported in the literature as adopting the locked-1 conformation (23, 38) (*SI Appendix*, Fig. S6).

### Bat SL-CoV WIV1 S Structural Features Recapitulate the Hallmarks of Prefusion State Locked-1 Conformation.

Structural criteria for an S glycoprotein in prefusion state to be designated as in locked conformation instead of in closed one are, i) the presence of lipids (e.g., LA) bound to the RBD FFABP of each monomer; ii) a cluster of symmetric H-bonds and salt bridges that keep the central helix (CH) and beta-hairpin (β-H) of the three monomers at the same reciprocal distance; iii) a rigidified CTD-3 with a highly disordered D-domain loop (D-loop); and iv) a highly ordered fusion peptide proximal region (FPPR) (23, 36, 37). In light of the similarities observed in the structural alignment, we wanted to analyze in more detail whether our bat SL-CoV WIV1 S was featuring the locked-1 conformation, and in what its conformation was differing from those of either previously determined WIV1 S structures, or best aligned locked-1 SARS-CoV1 and SARS-CoV-2 S. As first characteristic reminiscent of the locked-1 conformation, in our structure one LA molecule is bound to the FFABP of each S monomer through interactions of its aliphatic tail with 12 amino acids, nine of which are hydrophobic residues (Phe326, Val329, Ala351, Phe362, Phe365, Phe380, Val 383, Leu422, and Leu500) and three are polar ones (Cys324, Tyr353, and Tyr357) (*SI Appendix*, Fig. S7). Moreover, the carboxyl group of LA hydrophilic head establishes H-bonds with the side chain of Arg396 and Gln397, which are located in the RBD of the clockwise-adjacent (as seen from top view) S monomer, and serve as charged and polar anchors, respectively (*SI Appendix*, Fig. S2). Superposition to the other available LA-bound WIV1 S (PDB ID: 8TC0) and comparative analysis of LA-FFABP contacts within a 4 Å cutoff distance revealed that LA molecules accommodate differently in the FFABPs of the two structures, therefore their residue interaction patterns do not fully match (*SI Appendix*, Fig. S7). In particular, our LA does not bind to Ile346, Leu356, and Phe502, establishing instead unique interactions with Val329, Phe362, and Val383, and is anchored at much shorter distance to Arg396 (2.8 Å vs. 3.5 Å) and Gln397 (3.4 Å vs. 4.9 Å) than is the LA in the other model (*SI Appendix*, Fig. S7). Compared to structurally aligned orthologs from SARS-CoV-2 (PDB ID: 7OD3), SARS-CoV-1 (PDB: ID 8H0X) and civet SL-CoV 007 (PDB ID: 8TC1), to which our RBD is more similar, LA in our model is engaged in a higher number of interactions with the FFABP residues (*SI Appendix*, Fig. S6). Furthermore, LA is tightly juxtaposed in the FFABP of both WIV1 S structures, in such a way as to create minimal bulk, and very little difference (∼0.1 Å and 0.2 Å for our structure and PDB ID: 8TC0, respectively) exists in the D_pocket distance between Asn358 and Phe365—which demarcate the pocket entry—with respect to the FFABP of another WIV1 S structure (PDB ID: 8WQ0), in which an LA bound to the FFABP was not detected (*SI Appendix*, Fig. S7). Difference in the D_pocket distance between LA-bound and LA-free FFABP is much more pronounced in both SARS-CoV-1 (PDB ID: 8H0X, as compared to PDB IDs: 8H11 and 5XLR) and SARS-CoV-2 (PDB IDs: 7OD3, as compared to PDB IDs: 7XU3 and 6VXX) representative orthologs (*SI Appendix*, Fig. S8). Therefore, in light of the observed subtle differences in the D_pocket distance, and of the overall structural similarity with our model (RMSD 0.959 Å), it may be that also in the WIV1 S structure from Qiao and Wang (PDB ID: 8WQ0) there is LA bound to the FFABP, but that this ligand could not be reliably annotated because undetected due to poor density. For this reason, here we refer to this atomic model as LA-undetected rather than LA-free. As second feature ascribable to the locked-1 conformation, in our WIV1 S full symmetry is displayed in the cluster of H-bonds established between the carbon atoms of Arg1022 guanidine groups. An H-bond triplet keeps the CH (α14) and β-H (β53, β54) domains of each monomer close together around the S homotrimer central axis, with the three side chains of Arg1022 facing each other at the reciprocal distance of 4.5 Å, and is further stabilized by salt-bridges and π-interactions that each Arginine residue engages with Glu1014 and Phe1025, respectively, as well as by disulfide bridges between Cys1015 and Cys1026 (*SI Appendix*, Fig. S7). The values of these distances are consistent with those measured in the other LA-bound WIV 1 S (PDB ID: 8TC0), as well as in the structurally aligned SARS-CoV-2 and SARS-CoV-1 orthologs that showed the highest S2 similarity (PDB IDs: 7WGV and 8H0Y, respectively). Of note, the same distances in the LA-undetected WIV 1 S (PDB ID: 8QW0) are even shorter (∼4.4 to 4.3 Å) and slightly asymmetric (*SI Appendix*, Fig. S8). As third distinctive, combined sign indicating the adoption of the locked-1 conformation, in each monomer of our WIV1 S homotrimeric structure the D-loop (aa 603-628) is highly disordered (therefore is missing in the atomic model due to flexibility), whereas the FPPR is highly ordered and folded into the three helices α7, η3 and α8, the former and the latter being stabilized by a disulfide bridge between Cys823 and Cys834 (*SI Appendix*, Fig. S7). Consistently, among the structurally aligned LA-bound orthologs, three-α-helix folded FPPRs are also observed in each protomer of locked-1 SARS-CoV-2 (PDB ID: 7OD3) and civet SL-CoV 007 S (PDB ID: 8TC1), whereas in locked-1 SARS-CoV-1 (PDB ID: 8H14), LA-bound and LA-undetected WIV1 S (PDB ID: 8TC0 and 8WQ0, respectively) FPPRs are also ordered, however folded into two α-helices. By contrast, in locked-2 S from SARS-CoV-1 (PDB ID: 8H10) and SARS-CoV-2 (PDB ID: 7XU2) FPPRs are folded into one α-helix only, whereas in representative closed SARS-CoV-1 (PDB ID: 8H15), SARS-CoV-2 (PDB ID: 7XU3) and in open WIV1 S (PDB ID: 8WLY), FPPRs appear as folded into an extruded loop in the former, or as highly disordered in the two latter ones, respectively (*SI Appendix*, Fig. S8). Hence, not only the WIV1 S structure here described displays molecular signatures that are hallmarks denoting the locked-1 conformation, but also with its features it sufficiently differs from the other LA-bound and LA-undetected WIV1 S structures as to let inferring a unique metastable conformation for it. Consistently, analysis of the electrostatic surface potential shows markedly different patterns between the most similar SARS-CoV-1, SARS-CoV-2 and SL-CoV S to our structure, as well as between the three WIV1 S structures (*SI Appendix*, Fig. S9). In addition, further differences emerge between LA-bound and LA-free structures in regard to the compactness of the S homotrimer. In fact, between two SARS-CoV-1 S structures representative of the locked-1 and closed conformation (PDB IDs 8H0X and 8H11, respectively) it is observed that the three RBDs interact with each other through an extensive interface area in the former, whereas they are completely separated in the latter. For two representative locked-1 and closed SARS-CoV-2 S structures (PDB IDs 7OD3 and 7XU3, respectively), the RBDs keep in mutual contact in both conformations, although with a much less extensive interface area in the latter one. A similar pattern exists between LA-bound and LA-undetected WIV1 S (PDB IDs 8TC0 and 8WQ0, respectively), however with very little difference in the RBD interface area. Surprisingly, against this trend and despite being LA-bound itself, in our WIV1 S the interface area between RBDs is even lower than the one of its LA-undetected ortholog, thereby resulting as the least compact among the three WIV1 S homotrimeric structures so far determined (*SI Appendix*, Fig. S9).

### LA Dynamically Persists in Bat WIV1 S FFABP upon Complex Formation between RBD and ACE2 from Different Hosts.

**Fig. 2.**
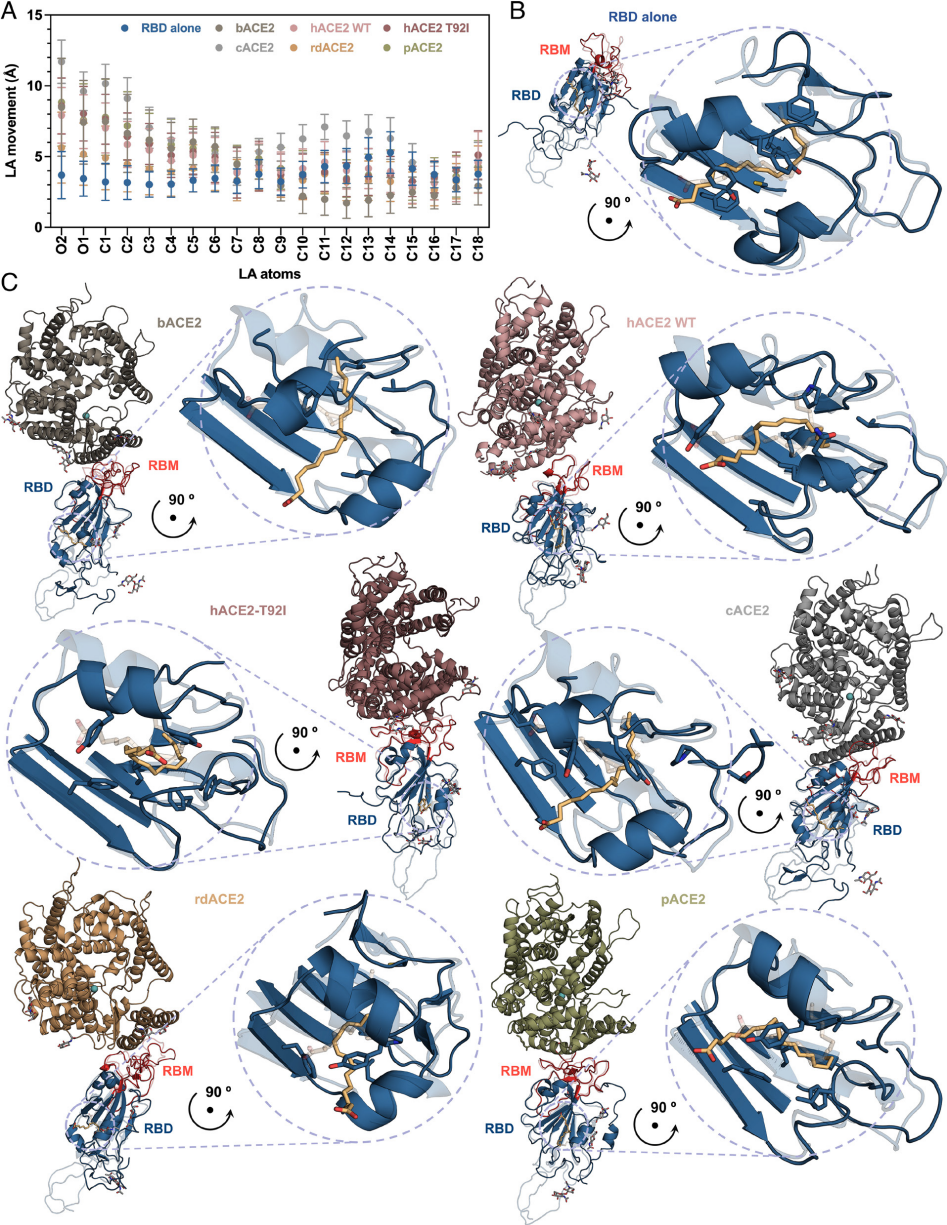
MD of LA repositioning within bat SL-CoV WIV1 S RBD-FFABP upon complex formation with ACE2 receptor. (*A*) Analysis of the movement of LA heavy atoms over the last 100 ns of the total (500 ns) MD simulation, calculated for the WIV1 S RBD alone (dark midnight blue) and in complex with bACE2 (rocket metallic), hACE2-WT (Tuscany pink), hACE2-T92I (copper rose), cACE2 (Spanish gray), rdACE2 (brown yellow), and pACE2 (moss green) receptors; values are plotted as mean and SD of experimental distribution over simulation time. (*B*) Ribbon representation of the WIV1 S RBD atomic model (RBM highlighted in deep carmine pink) from the cryo-EM structure presented in this work, shown after 500 ns MD simulation with FFABP highlighted by a dashed circle and as zoomed *Inset*; the same structure before MD simulation is also shown as transparent ribbon for comparison, highlighting the change of the LA position within FFABP at time frames. (*C*) Ribbon representation of the atomic model of cryo-EM WIV1 S RBD (this work) in complex with in silico modeled bACE2 (*Upper*-*Left*), hACE2-WT (*Upper*-*Right*), hACE2-T92I (*Middle*-*Left*), cACE2 (*Middle*-*Right*), rdACE2 (*Lower*-*Left*), and pACE2 (*Lower*-*Right*), shown after 500 ns MD simulation; the superposed atomic model of RDB alone before MD simulation is shown for comparison as transparent ribbon; the FFABPs by dashed circles and as zoomed *Insets*, and within them the LA position change at timeframes, are highlighted; LA molecules (peach orange) are shown as stick representation and colored by heteroatoms.

Because of the FFABP-bound LA, which keeps each RBD anchored to the clockwise-adjacent counterpart, the locked S homotrimer is less likely to transition into the open conformation. In line with this notion, not only biophysical experiments have shown that LA binds to SARS-CoV-2 S FFABP with very high affinity and slow off-rate but also MD studies have revealed that—although moving within it over simulation time—LA remains stably associated with the FFABP (21, 38). When removed from its binding site upon application of mechanical force, LA was shown to reinteract with the hydrophobic pocket, either in the RBD hidden in the “down” position of the locked conformation, or in the RBD exposed in the “up” position of the open one (36, 39). Also, persistence of the LA in the FFABP, change in its residue interactions and rebinding after removal from the pocket, were computationally observed in both the isolated RBD and the whole S homotrimer, in either SARS-CoV-1, Middle East respiratory syndrome (MERS)-CoV, and WT as well as variants of concern (VOC) SARS-CoV-2 systems, thereby suggesting a functionally meaningful and evolutionarily conserved role for LA in stabilizing the locked conformation (36, 40). Nevertheless, given that RBD exposure is a prerequisite for the RBM interaction with ACE2 and consequent virus entry into host cell, we reasoned that, if not the removal of LA from the FFABP, at least its repositioning within it, would be a required event for RBD unlocking and RBD-ACE2 complex formation. With this in mind, we asked ourselves whether for the locked-1 WIV1 S, and in the context of the RBD-ACE2 complex, LA would stay in the FFABP, and if it would reposition within it. We therefore performed comparative MD simulations on LA-bound WIV1 S RBD, either isolated or in complex with receptors from bat, civet, raccoon dog, pangolin, and human host. For the latter, it was reported that glycans attached to Asn90 interfere with binding to SARS-CoV-1 and SARS-CoV-2 RBD because of a steric hindrance at the interface between ACE2 and RBD (40, 41), and that disruption of N90 glycosylation by any mutation at this residue, or at adjacent ones forming the NxT/S glycosylation motif, would increase human ACE2 (hACE2) binding affinity for SARS-CoV-2 S RBD (42). Therefore, since in the hACE2 T92I polymorphism the lack of Asn90 glycosylation correlated with increased susceptibility to SARS-CoV-2 infection (43), in addition to WT hACE2 we also included this mutant to our computational analysis. Results showed that, over the course of 500 ns MD simulation and for all systems, LA never jumped out of the FFABP. Yet, it moved continuously within the pocket, with its unanchored hydrophilic head (O1, O2, and C1 atoms) exhibiting more extensive motion compared to the hydrophobic tail (C2-C18 atoms) (Fig. 2*A*). The movement of the LA head was more pronounced in all RBD-ACE2 complexes (about 8.0 ± 1.6 Å on average), than in the RBD alone (3.5 ± 1.5 Å). Overall, the RBD complexes exhibited comparable behavior across different host receptors, with the largest movement observed for the civet one (10.9 ± 1.6 Å). Furthermore, compared to the sole RBD (Fig. 2*B*), in the MD simulations of RBD-ACE2 complexes the LA decreased its interactions with FFABP residues while repositioning, with the lowest number of interactions observed for the complex with bat ACE2 (bACE2) and wild-type human ACE2 (hACE2). Intermediate values were found for the complexes with raccoon dog (rdACE2), pangolin (pACE2), civet (cACE2), and the human mutant with nonglycosylated Asn90 (hACE2-T92I) (Fig. 2*C* and *SI Appendix*, Fig. S10). Furthermore, quantitative analysis of the contact frequency (CF) during simulation time between LA and WIV1 S RBD, RBD-FFABP amino acid residues (and some from neighboring Domain C) heavy atoms at distance closer than 4.0 Å, revealed that residues with most frequent contacts with LA (i.e., for more than 80% frames out of last 100 MD simulation frames) vary between RBD alone and the complexes with ACE2 from different hosts (*SI Appendix*, Fig. S10). Specifically, in the isolated RBD, Leu356 and Phe365 ranked as the FFABP residues with most frequent contacts with LA. Upon RBD-ACE2 complex formation, Leu356 showed a lowered CF across all systems, whereas Phe365 exhibited the lowest CF in the complexes with bACE2, hACE2, and hACE2-T92I, while negligible differences were observed between the sole RBD and the complexes with rdACE2, pACE2, and cACE2 (*SI Appendix*, Fig. S10). In addition, while in the isolated RBD Tyr353, Leu356, and Phe365 maintain high CF with LA over all simulation time, only a few FFABP residues remain in closeness and frequent contact with LA in the context of the RBD-ACE2 complex, including Phe326 and Phe 365 for RBD-bACE2 and RBD-rdACE2, respectively; Tyr353 and Phe365 for RBD-pACE2; Ty353 and Ty357 for both RBD-hACE2-WT and RBD-hACE-T92I; Tyr353, Phe365, Cys367, and Leu516 for RBD-cACE2 (*SI Appendix*, Fig. S10). These results indicate that, when WIV1 S RBD interacts with ACE2, the presence of the latter is sufficient to determine repositioning of the LA molecule within the FFABP, in such a way that the S locked conformation is disfavored by loss of key LA-FFABP interactions, whereas the establishment of new interactions potentially favors the “unlocking” process. Transitions from a locked phase to an unlocked one compatible with interaction with ACE2, is most pronounced with human and bat receptor, whereas, for example, with the civet one the hydrophilic head of LA shows the greatest movement relative to RBD alone, likely due to instability in this intermediate host. Finally, the fact LA interaction with Leu356 and Phe365 significantly decreases in the transition from the locked phase to the unlocked one, suggests that these residues play a key role in stabilizing LA and therefore in the WIV1 S adoption of the locked-1 conformation.

### Transition of Bat WIV1 S RBD to Unlocked Conformation Depends on Extent of LA Repositioning within FFABP, Differing upon Binding to Diverse Host ACE2.

A distinctive phenotypic feature of bat SL-CoV WIV1 is the broad spectrum of hosts, as demonstrated by pseudotyped viruses (PVs) decorated with WIV1 S that were found able to infect mammalian cells expressing ACE2 from a wide array of animal species (32, 44). Moreover, the human receptor was the most efficient, even more than the one from the natural reservoir *R. sinicus* bat, in supporting the formation of membrane fusion-triggered syncytia, as well as WIV1 S PVs entry (30–32). Hence, in light of our finding of LA molecules stably bound to the RBDs of WIV1 S, and of the results from MD simulations of WIV1 S RBD-ACE2 complexes, we wanted to infer whether the observed differential repositioning of LA in the FFABP would have any impact on the RBD binding mode to ACE2. To this aim, we first computed for all systems the D_pocket at the end of MD simulation, whose fluctuations were reported as indicative of the RBD transition from locked to open conformation within the S prefusion state (36). Results showed that, as compared to the WIV1 S RBD alone, the D_pocket markedly decreases in the complexes with WT and T92I mutant hACE2, and so it does, even though to a lesser extent, in the RBD-bACE2 and RBD-pACE2 complexes. By contrast, D_pocket is minimally decreased and slightly increased in the RBD complexes with cACE2 and rdACE2, respectively (*SI Appendix*, Fig. S11). Notably, in the sole RBD, LA is compactly accommodated at the center of FFABP, with its hydrophilic head positioned deep inside. Conversely, in the RBD-bACE2, RBD-hACE2, and RBD-hACE2-T92I complexes, the LA hydrophilic head is positioned further outward from the pocket as compared to other systems (*SI Appendix*, Fig. S11), which coincides with the observed CF decrease for Phe365. In the RBD-cACE2 and RBD-rdACE2 systems, LA also exhibits a compact distribution, however in the former displaying a distinct binding location, which is evidenced also by the varied interacting residues in FFABP. Finally, in the RBD-pACE2 complex, LA displays a more disordered distribution (*SI Appendix*, Fig. S11). Overall, these results show that, compared to the RBD in the locked-1 conformation of the homotrimeric cryo-EM structure of WIV1 S, D_pocket reduction is observed in the isolated RBD (which is unbound to the adjacent RBD as in the transition to an unlocked conformation) as well as in most of the complexes with ACE2, and this is consistent with the significant decrease in interacting residues with LA. Nevertheless, the extent of D_pocket reduction, and therefore of unlocked conformation transition, varies among complexes with ACE2, suggesting differential evolutionary adaptation for the interaction with different host receptors.

### LA-Bound Bat SL-CoV WIV1 S RBD Dynamically Interacts with bACE2 and hACE2 More Stably and Strongly Than with Receptors from Potential Intermediate Hosts.

**Fig. 3.**
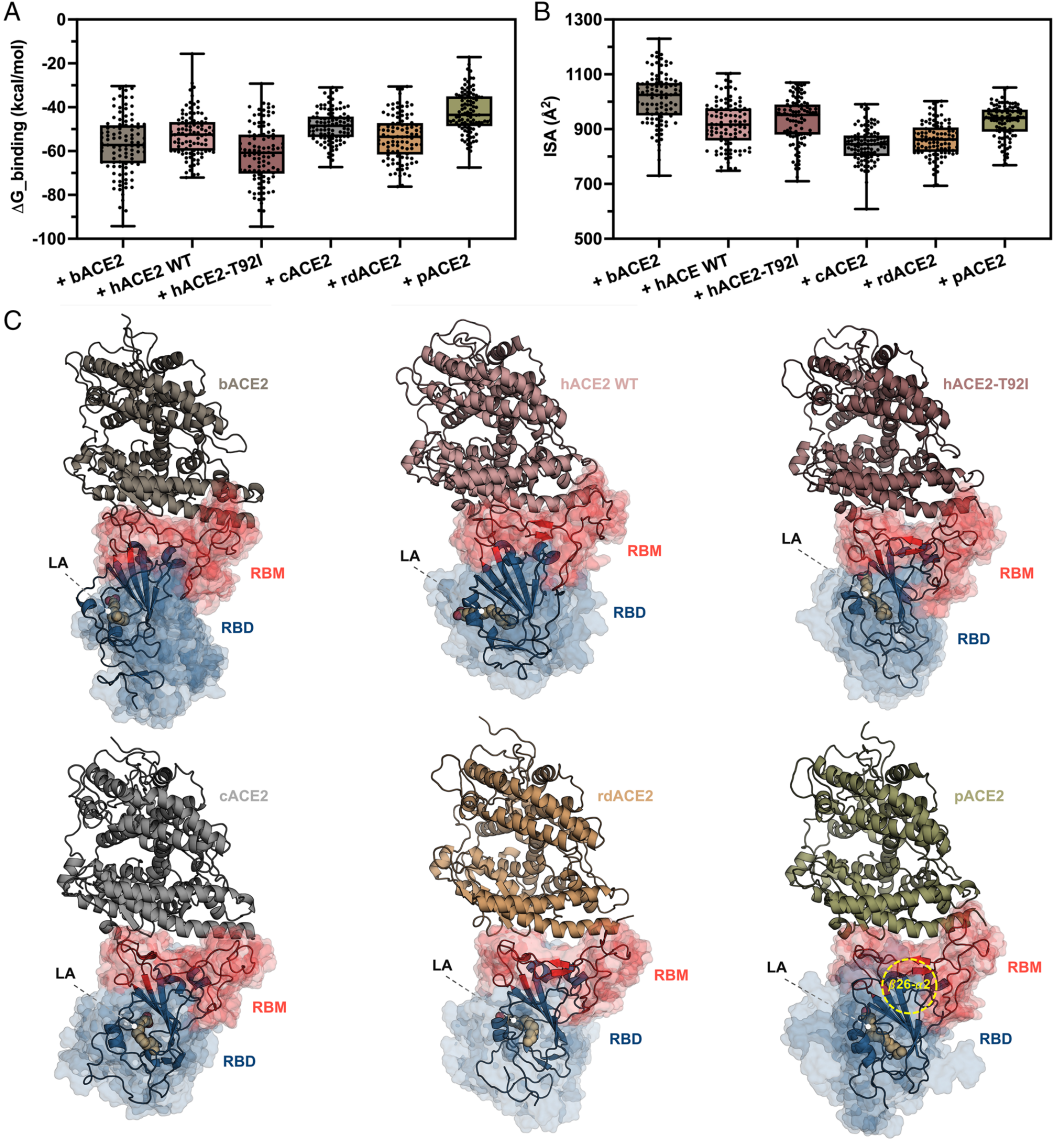
MD of the interaction of bat SL-CoV WIV1 S RBD with ACE2 receptors from different mammalian hosts. Analysis of (*A*) ΔG_binding and (*B*) ISA for the interaction between WIV1 S RBD with bACE2, hACE2-WT, hACE2-T92I, cACE2, rdACE2, and pACE2; the continuous line in the middle of each box represents the median value, with top and bottom boundaries of the box corresponding to the upper and lower quartiles, respectively, and error bar referring to the minimum and maximum values of the distribution (also shown as single experimental points). (*C*) Ribbon representation of AlphaFold3 generated complexes formed upon interaction of WIV1 S-RBD (dark midnight blue, with RBM highlighted in deep carmine pink) with bACE2 (*Upper* *Left*), hACE2-WT (*Upper* *Middle*), and hACE2-T92I (*Upper* *Right*), cACE2 (*Lower* *Left*), rdACE2 (*Lower* *Middle*), and pACE2 (*Lower* *Right*), as they appear at the end of 500 ns MD simulation; the dynamics of WIV1 S RBD conformations over MD simulation time is shown as transparent isosurface; LA (peach orange) is shown as sphere representation and colored by heteroatoms; β26-α2 region (aa 392-398) within WIV1 S RBD complexed to pACE2 that was highly dynamic over MD simulation time is highlighted by a yellow dashed circle. Color code for RBD and ACE2 from different hosts is the same as in Fig. 2.

Next, from the MD simulation trajectories, we computed the binding free energy (ΔG_binding) and the interacting surface area (ISA) at the interface between RBD and ACE2 for all systems, as measures of the interaction stability and closeness, respectively. Results showed that the highest amount of ΔG_binding is released when WIV1 S RBD forms a complex with bACE2 and hACE2-T92I, followed by the complex with hACE2-WT and rdACE2. Instead, the released ΔG_binding is of intermediate extent for the complexes with rdACE2 and cACE2 (Fig. 3*A*). Likewise, analysis of ISA resulted in a similar rank, in which RBDs complexed to bACE2 and both WT and hACE2-T92I, show the highest ISA values, whereas those complexed to rdACE2 and cACE2 have instead the lowest ISA (Fig. 3*B*). In contrast with the described ranking, the RBD-pACE2 complex displays the lowest ΔG_binding (Fig. 3*A*), nevertheless exhibiting an ISA of intermediate value (Fig. 3*B*), which indicates a condition where low stability and high closeness coexist. Of note, this—only apparent—discrepancy is indeed consistent with the behavior of a region, spanning between the β26 and α2 secondary structure elements of the RBD (aa 392-398), which was highly dynamic throughout the entire MD simulation, characterized by larger fluctuations in RMSD (*SI Appendix*, Fig. S12), suggesting that the binding mode of WIV1 S RBD to pACE2 slightly differs from that observed in the other complexes. Nonetheless, the ensemble conformations of all complexes are similar, and ACE2 always interacts with the RBM (Fig. 3*C*). Consistently, visualization of the footprint left by the interaction with ACE2 on WIV1 S RBD surface (and vice versa) as 2D projection by protein surface topography (PST) showed that residue contacts distribute on a wider area for the complex involving the bat receptor as compared to other hosts, and for the human mutant receptor as compared to WT (*SI Appendix*, Figs. S13 and S14). In this regard, it is worth noting that some interactions are observed in both complexes with WT and hACE2-T92I, such as the H-bonds established by Ser19 and Gln24 from hACE2 with WIV1 S RBD Asp464 and Tyr476, respectively. By contrast, other interactions such as a π–π interaction between Tyr41 on the receptor and Tyr492 on the RBD are exclusively present in the complex with hACE2-WT, whereas a salt bridge between Glu329 on the receptor and Arg427 on the RBD is uniquely observed in the complex with hACE2-T92I (*SI Appendix*, Fig. S15). These similarities and peculiarities highlight the interplay between evolutionary divergence and conservation as the forces shaping the interaction between WIV1 S RBD and host receptors. Taken altogether, these results suggest that the S RBD of bat SL-CoV WIV1 is still evolutionarily optimized for interaction with the ACE2 of its chiropteran natural reservoir, at the same time being able to interact with ortholog receptors from other potential intermediate hosts, albeit with less efficiency, and that, in addition, it is fully adapted to interact with both hACE2-WT and T92I polymorphism.

### Bat SL-CoV WIV1 S RBD Displays a Binding Affinity Gradient toward Different ACE2 within WIV1 Host Range Tropism.

**Fig. 4.**
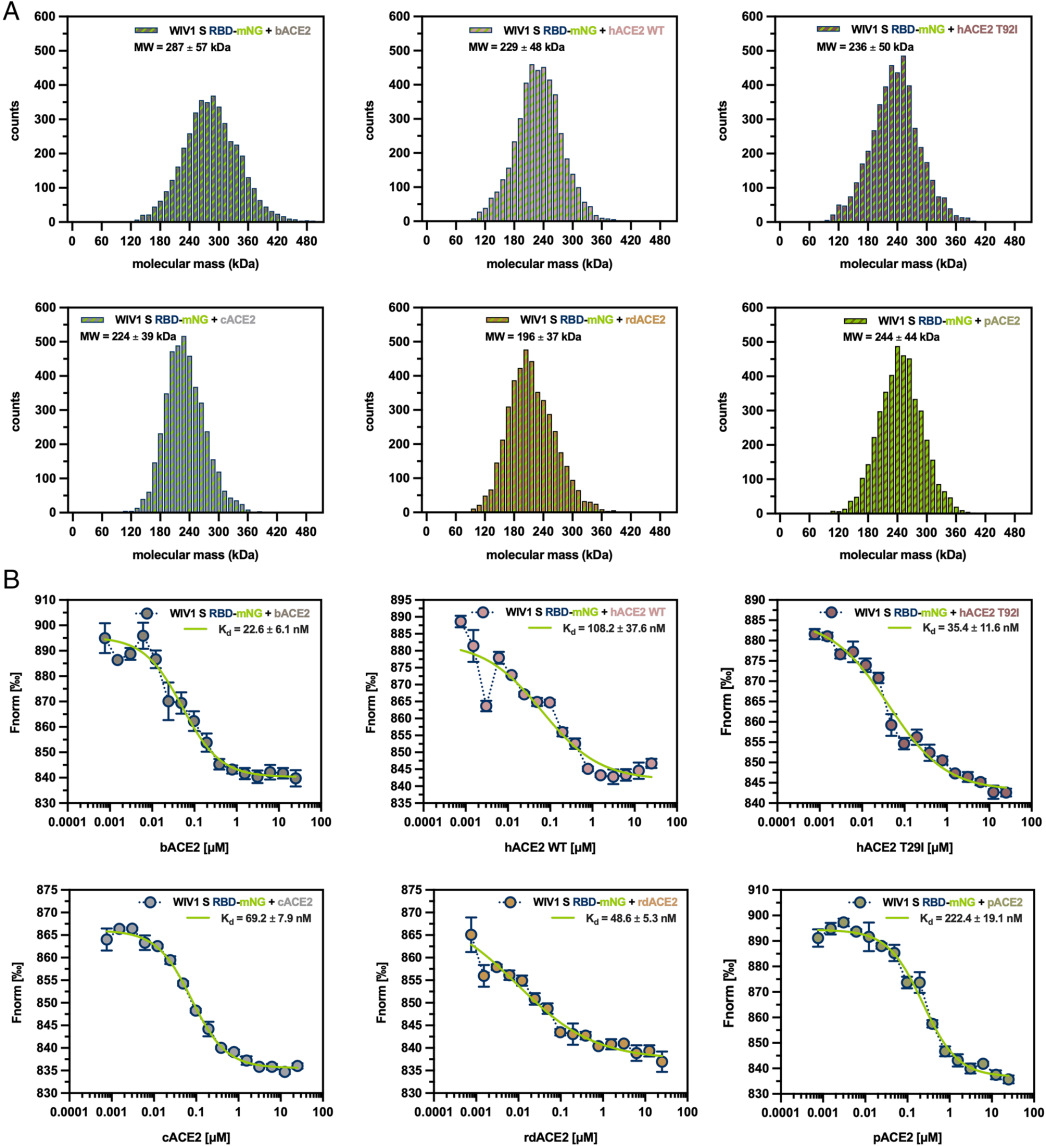
MP and MST analysis of the interaction of bat SL-CoV WIV1 S RBD with ACE2 receptors from different mammalian hosts. (*A*) MP histograms of molecular mass distribution showing the in-solution quaternary structure of the complex formed by WIV1 S RBD-mNG with bACE2 (*Upper* *Left*), hACE2-WT and hACE2-T92I (*Upper* *Middle* and *Right* panels, respectively), cACE2 (*Lower* *Left*), rdACE2 (*Lower* *Middle*), and pACE2 (*Lower* *Right*); mean and SD of MW values relative to each histogram peak are indicated. (*B*) MST binding affinity curves of the complex formed by WIV1 S RBD-mNG with bACE2 (*Upper* *Left*), hACE2-WT and hACE2-T92I (*Upper* *Middle* and *Right* panels, respectively), cACE2 (*Lower* *Left*), rdACE2 (*Lower* *Middle*), and pACE2 (*Lower* *Right*); connecting and nonlinear regression fitting curves on experimental points (circles) are represented as dotted (dark midnight blue) and continuous (sheen green) lines, respectively; experimental points and apparent K_d_ values are plotted and indicated, respectively, as mean and SD of at least four independent replicates. RBD and mNG are labeled in dark midnight blue and sheen green, respectively, whereas color code for ACE2 from different hosts is the same as in Fig. 2.

Given the results of differential ΔG_binding, ISA and PST values from computational analysis, we aimed at their validation through a biophysical characterization of the WIV1 S RBD-ACE2 interaction. Purified recombinant WIV1 S RBD fused to monomeric NeonGreen (mNG) fluorescent protein and ACE2 from the different hosts were mixed in the presence of an excess of LA and subjected to mass photometry (MP) and microscale thermophoresis (MST) analysis. MP analysis showed that WIV1 S RBD-mNG stably interacts in solution with all six ACE2 orthologs, with complexes displaying a MW of about 1.5 to 2.3 folds the expected theoretical 1:1 stoichiometry, possibly due to dimerization upon complex formation (Fig. 4*A* and *SI Appendix*, Fig. S16). Consistently, MST analysis detected WIV1 S RBD-mNG binding to ACE2 from all different hosts, showing apparent dissociation constant (K_d_) values in the low nanomolar range. Moreover, the highest binding affinities were measured between WIV1 S RBD-mNG and bACE2 or hACE2-T92I (about 23 nM and 35 nM on average, respectively), whereas K_d_ of intermediate values were measured for rdACE2, cACE2, and hACE2-WT (about 49 nM, 69 nM, and 108 nM on average, respectively) and the lowest binding affinity (≈222 nM on average) was measured for pACE2 (Fig. 4*B* and *SI Appendix*, Fig. S17). Taken together, results confirm that, on the one hand, WIV1 binds with high affinity to ACE2 receptors from different mammals, and that, on the other hand, can directly jump from the bat reservoir without requiring further adaptation through an intermediate host to spill over into the accidental human one.

## Discussion

Two decades have passed since the SARS-CoV-1 pandemic, 5 y now since the one caused by SARS-CoV-2, and throughout this time enormous efforts have been put toward an understanding of the molecular mechanisms underlying the infection by these viruses. Such efforts led to the validation of the S glycoprotein as the target of choice for therapeutics design, for which the ultimate trend is to warrant pan-coronaviral valence across Sarbecoviruses (45–48). Nevertheless, as both SARS-CoV-1 and SARS-CoV-2 belong to the same clade 1 encompassing all SL-CoVs known to infect humans, it is expectable that so-far unknown members from this group, or variants of known ones, will emerge in the future upon spillover and with the capability to escape existing countermeasures (1). Therefore, since it is mostly the S that undergoes mutations enabling to jump into the human population, information on the structural properties of this glycoprotein from any given SL-CoV with pandemic potential remains crucial. In this study, we provided an in-depth description of the S structure from the subclade 1a-belonging bat SL-CoV WIV1, which we captured by cryo-EM in a conformation resembling the locked-1 that was initially described for SARS-CoV-2 (21), and later also for SARS-CoV-1 S and MERS-CoV (36), remaining however poorly characterized for other SL-CoVs. As hallmark of locked conformation, an LA molecule present in each of the three RBDs prevents the adjacent RBD from folding outward as would happen in the transition from the closed to the open conformation. Noteworthy, the set of amino acids that in our WIV1 S RBD were found to interact with LA are highly conserved among SL-CoVs, suggesting that, in addition to their structural role in stabilizing the FFABP, they may have biological relevance in the virus life cycle, and future mutagenesis studies should be aimed at elucidating their function. In support of this notion, two of these residues, Arg396 and Gln397, are implicated in the recognition of WIV1 RBD as an antigen by the potent human monoclonal antibody 10-40 (33), further demonstrating that epitope concealment through the S-locked conformation induced by LA represents a key strategy for WIV1 to escape host immune response to viral infection. Indeed, as the main characteristic of locked homotrimeric S, LA is also present in the FFABP of the RBD of another WIV1 S structure (PDB ID: 8TC0), published while the writing of this manuscript was under way, however without any explicit contextualization in regard to a putative locked-1 conformation (28). Notwithstanding such similarity, and with the caveat of differences in density resolution, comparative structural analysis revealed substantial differences between these two WIV1 S structures, either in terms of LA position within the FFABP, as well as of interactions pattern of its heavy atoms with RBD residues, and overall conformation of the homotrimer. However, rather than being solely the reminiscence of a structural heterogeneity landscape, the two models suggest transitioning of WIV1 S across different metastable and isoenergetic subconformations, of which the one herein described fulfills all the structural hallmarks of a locked-1 S. Consistently, our cryo-EM structure also differs from a third WIV1 S published meanwhile, which is also in prefusion-state but devoid of annotated FFABP-bound LA (26), while showing the highest structural similarity with SARS-CoV-2 and SARS-CoV-1 orthologs denoted as in locked-1 conformation. On the one hand, as the result of LA functioning as locker that keeps the S RBDs in the “down” position, thereby avoiding their premature transition to the “up” one and their exposure for epitope recognition by neutralizing antibodies, the adoption of the locked-1 conformation confers selective advantage for immune evasion (23, 37). On the other hand, it was not fully clarified if, after repositioning to unlock the RBD, the LA would persist in the FFABP even during the RBD-ACE2 complex formation. Bats have long been considered the natural reservoir for SL-CoVs, with other wildlife potentially acting as intermediate hosts (49). With this notion in mind, we have undertaken a stepwise computational analysis of the LA behavior in the isolated WIV1 S RBD and in the context of its complexes with ACE2 across a panel of potential hosts. Our results from MD simulation show that, as previously observed for SARS-CoV-2 (39), also in the locked-1 WIV1 S RBD LA migrates to new positions within the FFABP to unlock the system, but also reveal that LA does so without detaching from the pocket even in the presence of an ACE2 molecule bound to the RBD. Moreover, decrease in the D_pocket distance as consequence of LA moving, well correlates with the virus evolutionary adaptation to its natural host, being such distance much lower for the WIV1 S RBD in complex with bACE2 from natural reservoir *R. sinicus* than for those with receptors from other mammals, which are therefore less likely to serve as optimal intermediate hosts. Together with the fact that LA repositioning and D_pocket distance decrease are even more pronounced during the complex formation between WIV1 S RBD and hACE2, our MD results are in line with virological observation that WIV1 is fully adapted for human infection (30, 31). Also, the efficiency of LA repositioning observed in WIV1 S RBD in the presence of hACE2 is particularly concerning in the event of a WIV1 variant—or a WIV1 lineage-related SL-CoV—to emerge, if it would bear mutations that render the unlocking process more rapid and easier. In this regard is worth noting that in the highly contagious SARS-CoV-2 VOC Omicron BA.2, an Arginine to Serine substitution at position 408 of S has weakened the H-bond mediated anchoring of LA hydrophilic head to the adjacent RBD. Likewise, in the S of SARS-CoV-2 VOC Delta plus B1.617.2-AY1, loss of positive charge due to mutation K417N determined decrease of inter-RBD stabilization (50). On its own, WIV1 S displays a Valine residue in the corresponding position 405, which in principle already favors, once the RBD is unlocked, its transition to the open conformation. Furthermore, MD results for the LA repositioning within FFABP are paralleled by those for the complex formation with ACE2. In fact, our findings show that the bat receptor exhibits the strongest binding for WIV1 S RBD, followed by the raccoon dog and civet ones, whereas the pangolin receptor is characterized by the highest dynamics and the weakest RBD binding. Notably, the differential binding of WIV1 S-RBD to ACE2 from different hosts from our in silico analysis was fully corroborated by experiments conducted under excess of LA, with MS and MST analysis describing the formation of stable complexes and interactions with nanomolar dissociation constants, respectively. Again, what emerged from the biophysical characterization is a binding affinity gradient of WIV1 S-RBD for ACE2 that, from the bat receptor, goes down through those of human with T92I polymorphism, raccoon dog, civet, human WT, and pangolin hosts. Thus, while suggesting that pangolins, well known for being hosts of clade 2 SL-CoVs (51–54) may be less suitable for the circulation of viruses from the WIV1 lineage, our data point back to the role played by bats in a spillover scenario involving WIV1 or a closely related SL-CoVs. Indeed, previous work demonstrated that—as compared to SARS-CoV-2—lower replication dynamics and weaker induction of proinflammatory response in human lungs during WIV1 infection may act as bottlenecks, rendering direct bat-to-human transmission very difficult (55). Moreover, while ecological studies have assessed as low the risk of direct viral transmission from bat to humans (56), and that in the case of WIV1 such risk may depend on the bat species harboring the virus (57), serological investigation conducted on people living in close proximity to Chinese caves inhabited by *R. sinicus* revealed retrospective human infections by WIV1-like bat SL-CoVs (58). Remarkably, in agreement with previous observation with PVs (32) and in line with our data on LA repositioning and D_pocket decrease, our MD simulation analysis of the S WIV1 RBD-ACE2 complex binding energy shows that WIV1 S is perfectly suited for interaction with the human receptor, even more efficiently, as compared to WT hACE2, in the case of the T92I polymorphism associated to severe Covid-19 (43). It is worth noting that, in SARS-CoV-1, S RBD adaptation to hACE2 was shown to be facilitated by the presence of an Asparagine at position 479 and a Threonine at position 487 (59). In the corresponding positions N480 and N488, WIV1 S is already human-adapted on the former site, whereas it still lacks the optimal residue on the latter one (44). Within this picture, it will be of paramount importance for surveillance of emerging WIV1 variants or WIV1-related SL-CoVs, to monitor for the rise of mutations at the S RBD that, like N488T, increase affinity with hACE2, especially if those will come in combination with residue substitutions that may destabilize the locked-1 conformation. In conclusion, the structural framework here presented adds up to the knowledge on elusive and metastable conformations adoptable by the coronaviral S glycoprotein and provides a picture on the dynamics by which an SL-CoV ready for human spillover such as WIV1 interacts with the entry receptor of its potential hosts. It is expected that such structural information will aid to foster prevention and response capabilities to mitigate the pandemic threat by SL-CoVs that might emerge in the future.

## Materials and Methods

Detailed description of molecular cloning, protein expression and purification, cryo-EM data collection and structure determination and analysis, MP and MST biophysical characterization, MD system setup, simulation parameters and analysis, are provided with relative references in *SI Appendix*.

## Supplementary Material

Appendix 01 (PDF)

## Data Availability

Atomic coordinates of the homotrimeric bat SL-CoV WIV1 S glycoprotein structure determined in this work have been deposited in the PDB with ID: 8ZMP (60), whereas the corresponding cryo-EM density map has been deposited in the Electron Microscopy Data Bank (EMDB) with accession code EMD-60253 (61). For any other structure used for comparative analysis, atomic coordinates were retrieved from the PDB, and their ID have been mentioned in the manuscript. All other data are included in the manuscript and/or *SI Appendix*.
