# Supplementary material for: Cryo-EM structure of locked spike glycoprotein from bat SARS-like coronavirus WIV1, molecular dynamics and biophysics across host range
Source: Proc Natl Acad Sci U S A. 2026 Feb 18;123(8):e2516874123. doi: 10.1073/pnas.2516874123 (PMC12933149; doi:10.1073/pnas.2516874123)
Supplement: Supplementary file 1 — Appendix 01 (PDF) [file pnas.2516874123.sapp.pdf]

PNAS

Supporting Information for

Cryo-EM structure of locked spike glycoprotein from bat SARS-like coronavirus WIV1, molecular dynamics and biophysics across host range

Chuan Liu [a, b, 1], Jingjing Zheng [a, c, 1], Yuhan Wang [a, c, 1], Florian Beck [d], István Nagy [b, e], Stefan Bohn [b, f], Jürgen M. Plitzko [d], Wolfgang Baumeister [a, b, *], Xiaoxiao Zhang [a, *], Liping Sun [a, *], Luca Zinzula [a, d, *]

[a] iHuman Institute ShanghaiTech University, 201210 Shanghai, China

[b] Max Planck Institute of Biochemistry, Emeritus Group Molecular Structural Biology, 82152 Martinsried, Germany

[c] School of Life Sciences and Technology, ShanghaiTech University, 201210 Shanghai, China

[d] Max Planck Institute of Biochemistry, Research Group CryoEM Technology, 82152 Martinsried, Germany

[e] Eszterházy Károly Catholic University, Center of Research and Development, 3300 Eger, Hungary

[f] CryoEM Platform and Institute of Structural Biology, Helmholtz Munich, 85764 Oberschleissheim, Germany

*To whom correspondence may be addressed: Luca Zinzula, Liping Sun, Xiaoxiao Zhang, Wolfgang Baumeister.

Email: zinzulal@shanghaitech.edu.cn; sunlp@shanghaitech.edu.cn; zhangxx4@shanghaitech.edu.cn; baumeist@biochem.mpg.de

[1] These authors contributed equally to the work.

This PDF file includes:

Materials and Methods
Table S1
Figures S1 to S17
References

Materials and Methods

Molecular cloning, protein expression and purification. Codon-optimized cDNA encoding for bat SL-CoV WIV1 S (GenBank accession code AGZ48831.1) was obtained from synthetic preparation (BioCat), and subcloned into pFastBacI vector under the restriction sites *Bam*HI and *Hind*III for the expression in Sf9 insect cells of a recombinant version of the WIV1 S ectodomain (residues 20-1194) as secreted protein. Baculovirus-infected Sf9 cells were cultured in 2L of EX-CELL 420 serum free medium (Sigma-Aldrich) for 72 h at 121 rpm at 26 °C, and harvested by centrifugation at 4500 × g at 4 °C for 30 min. Purification by immobilized metal ion AC (IMAC) via the protein C-terminal hexahistidine tag (6His-tag) was then performed by loading the 0.45 μm filtered cell culture supernatant onto a 5mL Ni-INDIGO Compact Cartridge (Cube Biotech) resin chromatographic column equilibrated in Buffer A (50 mM Sodium Phosphate, pH 8.0; 500 mM Sodium Chloride; 10% Glycerol; 10 mM Imidazole) and connected to a fast protein liquid chromatography (FPLC) ÄKTA pure™ protein purification system (Cytiva). After washing with 10 × column volume (CV) Binding/Washing Buffer A, protein was eluted in Elution Buffer B (Buffer A supplemented with 250 mM Imidazole), then concentrated with a 10,000 Da molecular weight cutoff (MWCO) Amicon® Ultra 15 centrifugal filter (Merck Millipore) at 3700 × g and 4 °C, up to ~ 3 mg mL-1, for subsequent SEC purification on a FPLC-connected Superdex 200 10/300 GL increase column (Cytiva) equilibrated in SEC Buffer C (25 mM Tris-HCl, pH 7.5; 150 mM Sodium Chloride). SEC elution peak fraction was selected, analysed by SDS-PAGE in 1X MES Running Buffer, 12% Bis-Tris Nu-PAGE™, PageRuler™ Prestained Protein Ladder (ThermoFisher Scientific) for target protein confirmation and immediately used for subsequent cryo-EM experiments. Codon-optimized versions of the cDNA encoding for bat SL-CoV WIV1 S RBD (GenBank accession code AGZ48831.1; aa 307-528) C-terminally fused to mNG (GenBank LC008486.1; aa 1-236) and bACE2 (*R. sinicus*; GenBank KC881004.1; aa 1-615), hACE WT and T92I (*H. sapiens*; GenBank NM_001371415.1; aa 1-615), cACE2 (*Paguma larvata*; GenBank AY881174.1; aa 1-615), rdACE2 (*Nyctereutes procyonoides*; GenBank EU024940.1; aa 1-614) and pACE2 (*Manis javanica*; GenBank XM_017650263.3; aa 1-615) were obtained from synthetic preparations (Azenta Life Sciences) and subcloned into pCAG vector under *Kpn*I and *Xho*I restriction sites, amplified in *Escherichia coli* DH5-α cells (Vazyme) and purified using GoldHi EndoFree Plasmid Maxi Kit (CWBio) following manufacturer instructions, for subsequent gene expression in Expi293F™ (ThermoFisher Scientific) human cells as recombinant C-terminally octahistidine (8His)-tagged secreted proteins. Cells were transfected at 2×10^6 cells mL^{-1} density using

polyethylenimine (PEI) Max (Polysciences) with 2 μg plasmid DNA mL^{-1} culture following manufacturer instructions, then cultured in Union293 (UnionBiotech) medium at 125 rpm, 30 °C, and after 66 hours separated from culture medium by centrifugation for 20 min at 4000 rpm, 4 °C. Supernatant was supplemented with 5 mM Calcium Chloride, 5 mM Magnesium Chloride, 1 mM Nickel Chloride and 0.2 mM Zinc Chloride, then loaded on an Econo-Pac® chromatography column (Biorad) packed with 1 mL Ni Sepharose™ High Performance IMAC resin (Cytiva) equilibrated in Binding Buffer D (25 mM HEPES, pH 8.0; 300 mM Sodium Chloride; 5 mM Calcium Chloride, 5 mM Magnesium Chloride, 1 mM Nickel Chloride; 0.2 mM Zinc Chloride; 5% Glycerol; 10 mM Imidazole) supplemented with 1X EDTA-free Protease Inhibitor Cocktail (APExBIO). IMAC purification was performed by flow gravity in Washing Buffer E (Buffer D supplemented with 30 mM Imidazole) and Elution Buffer F (Buffer D supplemented with 900 mM Imidazole). Subsequent SEC purification was performed on a Superdex 200 Increase 10/300 GL column (Cytiva) connected to an ÄKTA pure™ FPLC system (Cytiva) and equilibrated in SEC Buffer G (25 mM HEPES, pH 7.4; 150 mM Sodium Chloride; 5 mM Calcium Chloride, 5 mM Magnesium Chloride, 1 mM Nickel Chloride; 0.2 mM Zinc Chloride; 2.5% Glycerol). SEC-elution peak fractions were analysed by SDS-PAGE in 1X MOPS Running Buffer, 15% Bis-Tris Sure-PAGE™(Genscript), ColorMixed Protein Marker (Solarbio) for target protein confirmation, and positive fractions were pooled for concentration with a 10,000 Da MWCO Amicon® Ultra 15 centrifugal filter (Merck Millipore) at 3700 × g and 4 °C, up to ~ 5 and ~ 4 mg mL^{-1} for RBD-mNG and ACE2, respectively, then flash-frozen in liquid nitrogen and stored at -80 °C until use in subsequent experiments.

Cryo-EM sample preparation and data collection. Purified SL-CoV WIV1 S (3.8 μL at ~ 0.7 mg mL^{-1}) was applied to Copper 100 Holey Carbon Films 200 mesh R 1.2/1.3 EM grids (Quantifoil), glow discharged for 20 s in air using a PDC-32G Plasma Cleaner (Harrick Plasma) set to medium power, and vitrified in liquid ethane-propane by using a Vitrobot Mark IV (ThermoFisher Scientific) plunge-freezing system after blotting on filter paper for 2.5 s at 4 °C and 95 % humidity, with force value set to 0. Cryo-EM SPA data was acquired on a FEI Titan Krios transmission electron microscope (ThermoFisher Scientific) using the SerialEM (1) software package. Movie frames were recorder at a nominal magnification of 29,000 X using a K3 Summit direct electron detector (Gatan). The total electron dose of ~ 60 electrons per Å2 was distributed over 30 frames at a calibrated physical pixel size of 1.09 Å. Cryo-EM micrographs were recorded in a defocus range of -0.5 to -3.0 μm. Micrographs were

processed on the fly using the Focus (2) software package, if they passed the selection criteria, i.e., iciness < 1.05 Å; drift 0.4 Å < x < 70.0 Å; defocus 0.5 μm < x < 5.5 μm; estimated contrast transfer function (CTF) resolution < 6.0 Å. Micrograph frames were aligned using MotionCor2 (3) software and the CTF for aligned frames was determined using GCTF (4) software.

Cryo-EM image processing, 3D reconstruction, model building and refinement. Motion-corrected frames of the 2,645 collected micrographs were used for SPA processing using CryoSPARC (5). First, CTF parameters were estimated by Patch CTF, then particle auto-picking was performed using Blob Picker and Topaz Picker (6). A total number of 654,162 particles was picked and screened by several rounds of 2D classification. Next, the kept 120,508 particles were subjected to a round of 3D heterogeneous refinement, after which the selected 91,339 particles underwent 3D homogeneous and non-uniform refinement (7). Local resolution distribution was evaluated (8), and final average resolution was estimated to 3.38 Å according to the gold-standard criterion of Fourier shell correlation (FSC) = 0.143 by using the built-in tool in CryoSPARC (9). To build the atomic model of the WIV1 S glycoprotein homo-trimer, the cryo-EM structure of SARS-CoV-1 S in pre-fusion state (PDB ID: 6ACC) (10) was used as template for sequence alignment based on Sculptor in PHENIX (11). Next, the obtained output was used for fitting into the cryo-EM density of the ternary WIV1 S by using Chimera (12), and manually adjusted in COOT based on the experimental cryo-EM density map (13). After adjusting residues coordinates, one molecule of LA ligand (PubChem CID: 5280450), was fitted into the corresponding density within each RBD, whereas Asparagine-linked glycans were manually added to the corresponding densities. The fitted model was refined against the cryo-EM density map using real space refinement with secondary structure and geometry restrains in PHENIX (11). The final model after refinement was validated using MolPorbity and EM Ringer built-in tools in the PHENIX package (11). The model statistics is summarized in *SI Appendix*, Table 1.

MD simulations. *Systems setup.* Structures of bat SL-CoV WIV1 S RBD complexed to ACE2 from five different susceptible host species, namely Chinese rufous horseshoe bat (*R. sinicus*; GenBank: AGZ48803.1), human (*H. sapiens*, GenBank: NP_001358344.1), masked palm civet (*P. larvata*; GenBank: AAX63775.1), raccoon dog (*N. procyonoides*, GenBank: ABW16956.1) and Malayan pangolin (*M. javanica*, GenBank: XP_017505752.1) were prepared. For those species lacking an

experimentally determined ACE2 native structure in the PDB (i.e., bat and raccoon dog), consensus alignment of aa sequence spanning residues 19-615 (bat numbering) was chosen as input for template-free ab initio modelling of their atomic structure by AlphaFold 3 (14), whereas for the other ones (i.e., civet, PDB ID: 7WSK; pangolin, PDB ID: 7DHX; human, PDB ID: 6ACG), monomeric ACE2 structures were isolated from the retrieved PDB files (10, 15, 16). Moreover, in light of the reported modulatory effects of ACE2 glycosylation on RBD binding, receptors were arbitrarily glycosylated across all species (15-19) at sites determined based on corresponding templates (PDB IDs: 7C8K, 8VQR, 7WSK, 7DHX and 2AJF for bat, raccoon dog, civet, pangolin and human ACE2, respectively) using Maestro software (Schrödinger, LLC, 2024). For the human host, the receptor was prepared both as glycosylated at Asn90, or non-glycosylated due to a Threonine-to-Isoleucine mutation at position 92 (20). Next, WIV1 S RBD-ACE2 complexes for the various host species were generated based on best-ranked structural templates (PDB IDs: 7C8J, 8VQR, 7WSK, 7DHX and 6ACG for bat, raccoon dog, civet, pangolin and human ACE2, respectively) by using Swiss-Model server (21). Onto these homology-modelled backbones, our cryo-EM determined WIV1 S RBD (chain B, aa 318-514) and the PDB-retrieved or AlphaFold-modelled ACE2 were both superposed and structurally aligned using the Match-Maker tool built in Chimera software (22). Each RBD-ACE2 complex was then solvated in a cubic box with TIP3P water (23), including a 14 nanometer (nm) padding on all sides. Sodium (Na+) and chloride (Cl-) ions were added to achieve 150 mM physiological concentration, compensating for the proteins net charge. Finally, all six RBD-ACE2 configurations were energy-minimized using the steepest descent algorithm. *Simulation details.* Atomic MD simulations were conducted using GROMACS 2024.1 (24) with a time step of 2.0 fs. The CHARMM36 force field (25) was used for the proteins, with glycan parameters generated using CHARMM-GUI (26). The length of relevant disulfide bridges was set to 2.05 Å. The systems were first equilibrated using the Nose-Hoover integrator in the NVT ensemble (p = 1 atm, T = 310 K) for 500 ps with a timestep of 1.0 fs. Production runs were performed for 500 ns each in the NPT ensemble. Electrostatic interactions were computed using the Particle Mesh Ewald (PME) method with a 1.2 nm cutoff, while van der Waals interactions were calculated using the Verlet list scheme with a switching cutoff between 1.0 and 1.2 nm. A linear bond constraint solver (LINCS) algorithm was used to constrain the bond lengths involving hydrogen atoms (27). Periodic boundary conditions were applied in all three dimensions to avoid boundary effects caused by a finite simulation system. *Analysis.* The final 100 ns of simulation trajectories were selected based on relatively small RMSD value of 1.5-2.5 Å

for the backbone atoms of the protein complex. Analysis was performed using GROMACS utilities, MM/PBSA for binding free energy calculations (28), and custom scripts for distance measurements, alongside previously published scripts for specific interactions (29). The RBD pocket entrance distance, denoted as D_pocket, was computed as the distance between C-α atoms of residues Asn358 on the η1 helix and Phe365 on the β25 strand, forming the pocket entrance.

MP analysis. MP measurements were performed on a TwoMP (Refeyn) instrument, set up to monitor protein landing events to regular field of view (i.e., 10.9 × 4.3 μm^2) as detection area, 475.2 Hz frame rate and 60 s acquisition time, and operated using the AcquireMP (Refeyn) software and MassGlass™ UC Sample Prep Kit (Refeyn) ready-to-use six-well glass slides. Instrument calibration was performed using thyroglobulin and β-amyloid mouse monoclonal antibody (Sigma-Aldrich). For controls, purified WIV1 S RBD-mNG and ACE2 recombinant proteins were pre-diluted to 40 nM in SEC Buffer G, then measured at 20 nM final concentration in 20 μL of a 1:1 dilution with Buffer G. For assessment of RBD-ACE2 complex formation, pre-diluted 80 nM WIV1 S RBD-mNG and ACE2 from different hosts were mixed to 1:1 molar ratio and incubated at RT for 5 min, then mixture was diluted as for the isolated controls, in 20 μL Buffer G to 20 nM final concentration. Raw data were converted into mass distribution histograms, analyzed using the DiscoverMP (Refeyn) and plotted with GraphPad Prism (https://www.graphpad.com) software.

MST analysis. MST measurements were performed on a Monolith NT.115 (NanoTemper Technologies) instrument set up to Nano-blue channel (460-490 nm excitation wavelengths), 23 °C, 70% light-emitting diode (LED) and high MST power, respectively, and operated using the PR.ThermControl software (NanoTemper Technologies). Purified WIV1 S RBD-mNG and ACE2 were diluted in MST Buffer H (Buffer G supplemented with 0.05% Tween-20 and 0.1 μM LA), mixed and incubated for 1 hour at room temperature (RT), then loaded on standard capillaries (NanoTemper Technologies) for measurement. Binding affinity assay reactions were set up over sixteen experimental points by two-fold serial dilutions at RBD-mNG and ACE2 final protein constant concentration of 0.02 μM and concentration range of ~ 0.0008-25 μM, respectively. For each MST trace corresponding to a given ligand (i.e., ACE2) concentration, the heated state relative fluorescence (F_1, hot; 4.0-5.0 s) was divided by the cold state one (F_0, cold; -1-0 s) to obtain the corresponding normalized fluorescence

(Fnorm) ratio. Fnorm values expressed as part per thousand (‰) were plotted as function of ligand concentration to obtain dose-response MST curves. Data from at least four independent measurements representative of the MST trace fluorescence signal at 5.0 s (MST-On time) were averaged as mean ± standard deviation values, using the MO. Affinity Analysis (NanoTemper Technologies) and GraphPad Prism (https://www.graphpad.com) software for apparent K_d calculation and plotting, respectively. Statistical significance was evaluated by one-way analysis of variance (ANOVA) coupled to post-hoc Tukey test for multiple comparisons.

Structural bioinformatics and molecular graphics. Molecular visualizations were performed using PyMOL (Schrödinger, LLC, 2015), Chimera (12) and Chimera X (30) software. Color names were assigned based on hexadecimal codes as defined in the SchemeColor database (https://www.schemecolor.com). Annotation of glycosylation sites and of secondary structures topology was performed using PDBsum (31) server. Multiple sequence alignment (MSA), building of phylogenetic tree and mapping of MSA on the secondary structure topology were performed using Clustal Omega (32) server, FigTree (http://tree.bio.ed.ac.uk/software/figtree) software and ESPript (33) server, respectively, with aa sequences retrieved from the National Center for Biotechnology Information (NCBI) Protein database, namely bat SL-CoV WIV1 (GenBank: AGZ48831.1), and RsSCH014 (GenBank: AGZ48806.1), SARS-CoV-1 Tor2 (GenBank: YP_009825051.1), civet SL-CoV 007 (GenBank: AAU04646.1) and SZ3 (GenBank: AY304486.1), pangolin SL-CoV GD (QIG55945.1) and GX (QIA48614.1), bat SL-CoV RaTG13 (GenBank: QHR63300.2), BANAL-20-236 (GenBank: UAY13253.1) and 20-52 (Genbank: UAY13217.1), and SARS-CoV-2 (GenBank: YP_009724390.1). Structural alignments, superposition of atomic models and their fitting into cryo-EM density maps were carried out using MatchMaker, Match Align and fit-in-map tools built in Chimera software, respectively (22, 30). Calculation of the Poisson-Boltzmann electrostatic surface potential was performed using the PARSE force field in PDB2PQR (34) and APBS (35) software implemented as built-in tools in PyMOL (Schrödinger, LLC, 2015). For the mapping of residue contacts in static cryo-EM derived atomic models, COCOMAPS (36) server was used with a 4.0 Å cutoff of interaction distance. For analysis of surface topography, the PST server was used (37) on the Galaxy platform (38), with default parameters exception made for the setting of a 4.0 Å interaction distance cutoff. All plots were made using GraphPad Prism (https://www.graphpad.com) software.

Supporting Table 1. Cryo-EM data collection, refinement and validation statistics.

Entry name	WIV1 spike glycoprotein in locked state
**PDB ID; EMDB ID**	**8ZMP; EMD-60253**
**Data collection**	
Cryo electron microscope	FEI Titan Krios
Voltage (kV)	300
Detector	Gatan K3 Summit
Energy filter slit width (eV)	20
Magnification	29,000 ×
Pixel size (Å)	1.09
Total electron exposure (e^{-}/Å^{2})	60
Number of frames collected	32
Exposure rate (e^{-}/pixel/sec)	10
Defocus range (μm)	-0.5 ~ -3.0
Automation software	Focus v1.0.0
Micrographs collected	2,645
**3D reconstruction**	
Software	CryoSPARC v4.5.1
Total extracted particles	654,162
Total number particles for final refinement	91,339
Symmetry imposed Resolution range (Å)	C3
Resolution (Å) after refinement (FSC=0.143)	3.38
Map sharpening B-factor (Å^{2})	-60
**Refinement and validation**	
Software	Phenix.real_space_refine (1.21)
Model resolution (Å)	3.4
Model composition	
Non-hydrogen atoms	26,175
Protein residues	3,198
Map-model CC (overall/local)	0.80
B factors (Å^{2})	10,737
**R.M.S deviations**	
Bonds lengths (Å)	0.006
Bonds angles (°)	1.003
MolProbity score	2.29
Clash score	7.35
Rotamer outliers (%)	5.41
Cβ outliers (%)	0.10
CaBLAM outliers (%)	3.43
EMRinger score	3.99
**Ramachandran plot statistics**	
Preferred (%)	95.31
Allowed (%)	4.69
Outlier (%)	0.00

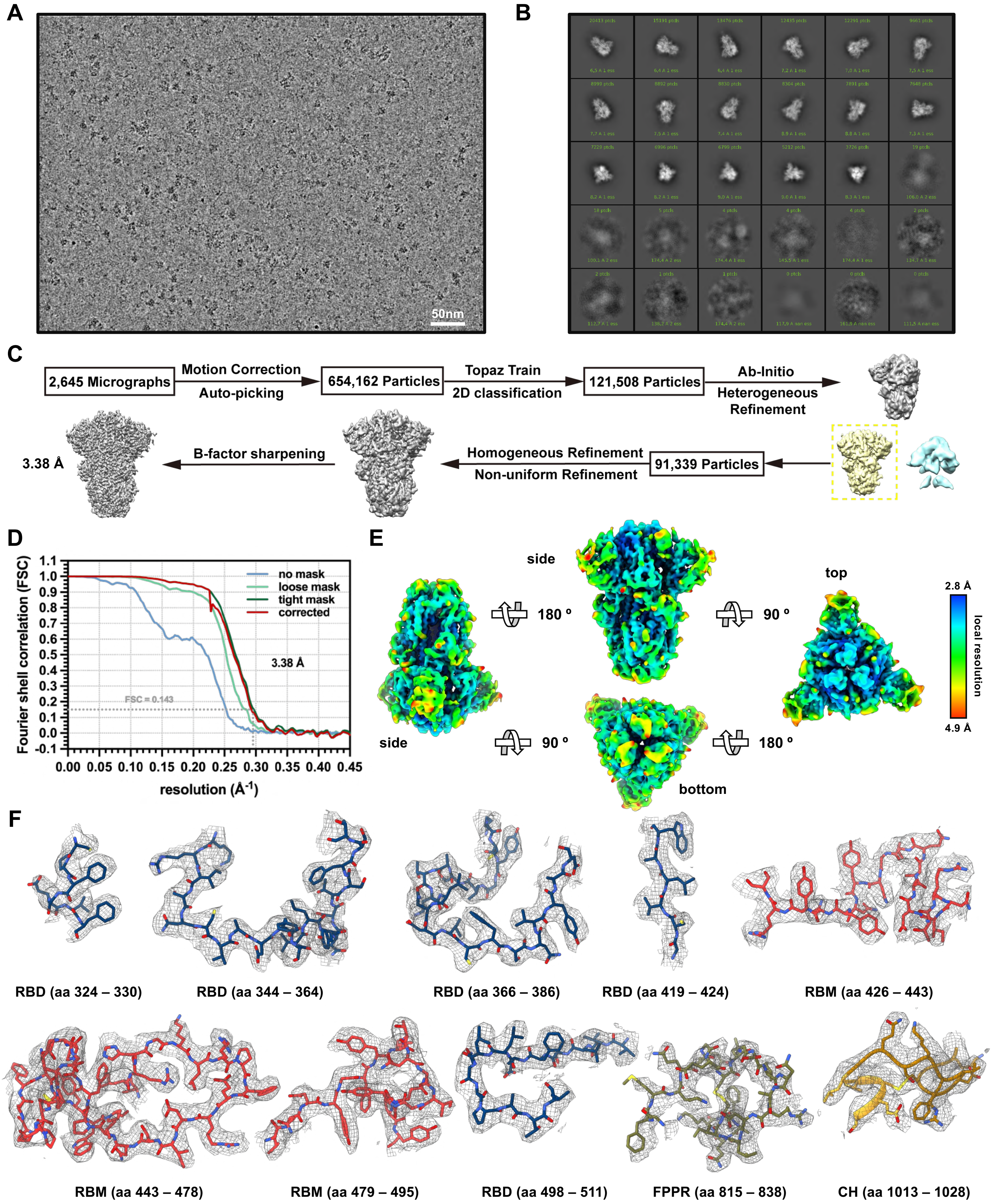

Fig. S1. Structure determination of bat SL-CoV WIV1 S by cryo-EM SPA. (*A*) Representative motion-corrected cryo-EM micrograph of the image dataset (n=2,645 micrographs) of the vitrified sample of purified bat SL-CoV WIV1 S (scale bar, 50 nm). (*B*) Representative 2D class averages of pre-fusion state WIV1 S from the reference-free 2D classification step of SPA workflow. (*C*) Scheme of the SPA image processing workflow for the 2D classification, 3D reconstruction and 3D refinement of WIV1 S cryo-EM density map. (*D*) Gold-standard Fourier shell correlation (FSC) curves for the cross-validation between unmasked, masked and corrected cryo-EM density maps of bat SL-CoV WIV1 S; the dotted gray line refers to the threshold cutoff of FSC = 0.143 at which final resolution of 3.38 Å was determined. (*E*) Local resolution estimate for the final cryo-EM density map of WIV1 S, shown as isosurface representation in four orthogonal orientations, rainbow-colored according to a resolution heatmap spanning 2.8-4.9 Å. (*F*) Representative densities and fitted built atomic models, shown as gray mesh and colored ribbon cartoons, respectively, of WIV1 S RBD (dark midnight blue) and CH (satin sheen gold) domains, and of RBM (deep carmine pink) and FPPR (mustard green) subdomains.

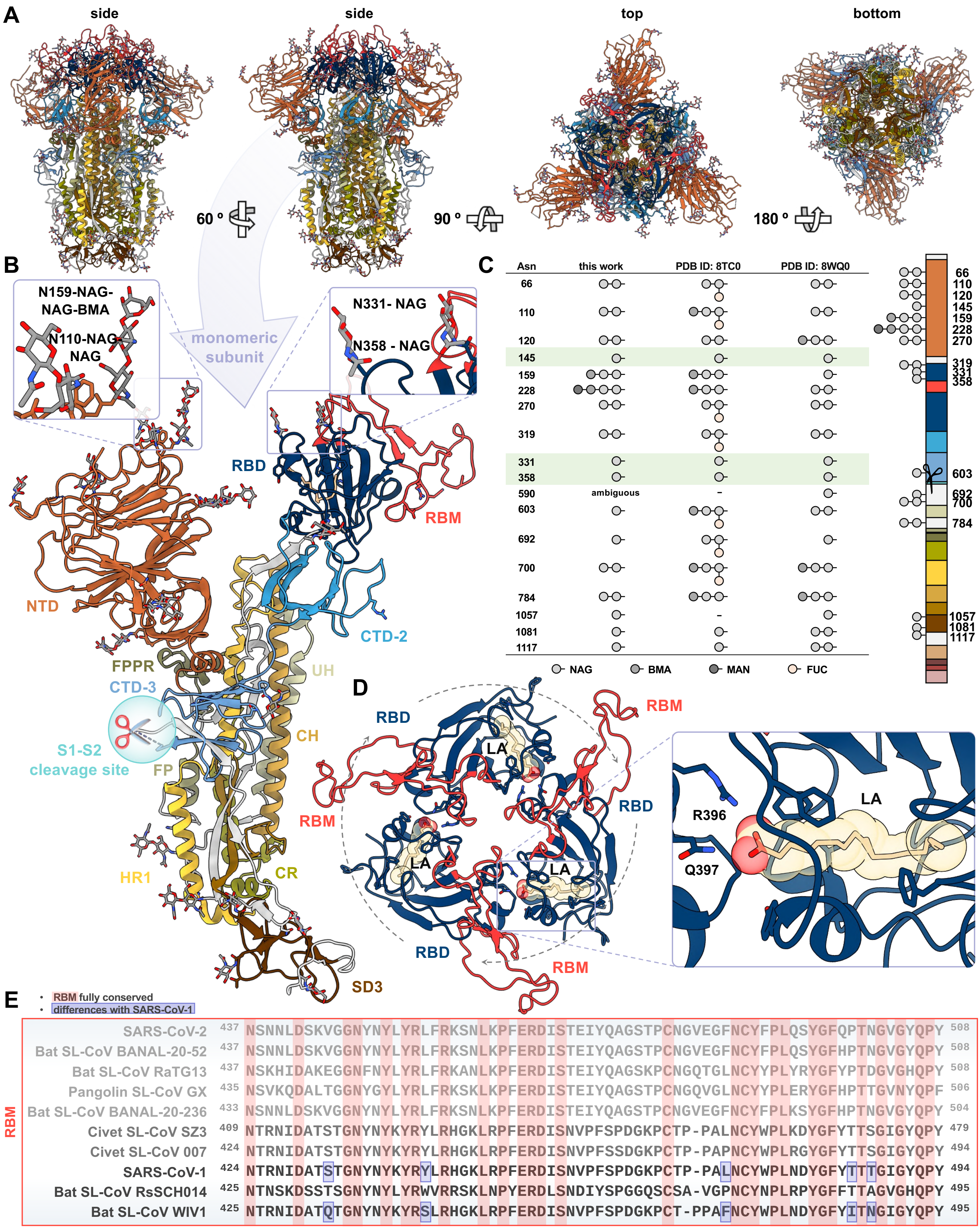

Fig. S2. Atomic model of locked-like bat SL-CoV WIV1 S. (*A*) Ribbon representation of homo-trimeric bat SL-CoV WIV1 S ectodomain, shown in four orientations; glycosylated residues are represented as sticks. (*B*) Ribbon representation of monomeric WIV1 S in side view orientation; domains and representative glycosylation sites are highlighted in different colors and as zoomed insets, respectively. (*C*) Comparative table (left panel) of glycosylation profiles of WIV1 S (this work) and previously determined structures (PDB ID: 8TC0; 8WQ0), with identical patterns highlighted in Chinese white background; cartoon of glycosylation sites (right panel) mapped on WIV1 S structure (this work) color-highlighted domains. (*D*) Ribbon representation of homo-trimeric WIV1 S RBD (dark midnight blue) and RBM (deep carmine pink) in top view orientation; sphere-stick represented LA bound to FFABP and clockwise-adjacent RBD is highlighted as zoomed inset. (*E*) Amino acid multiple sequence alignment of representative SL-CoVs S RBM; residues fully conserved and differing between WIV1 and SARS-CoV-1 are highlighted with deep carmine pink background and boxed in slate blue, respectively.

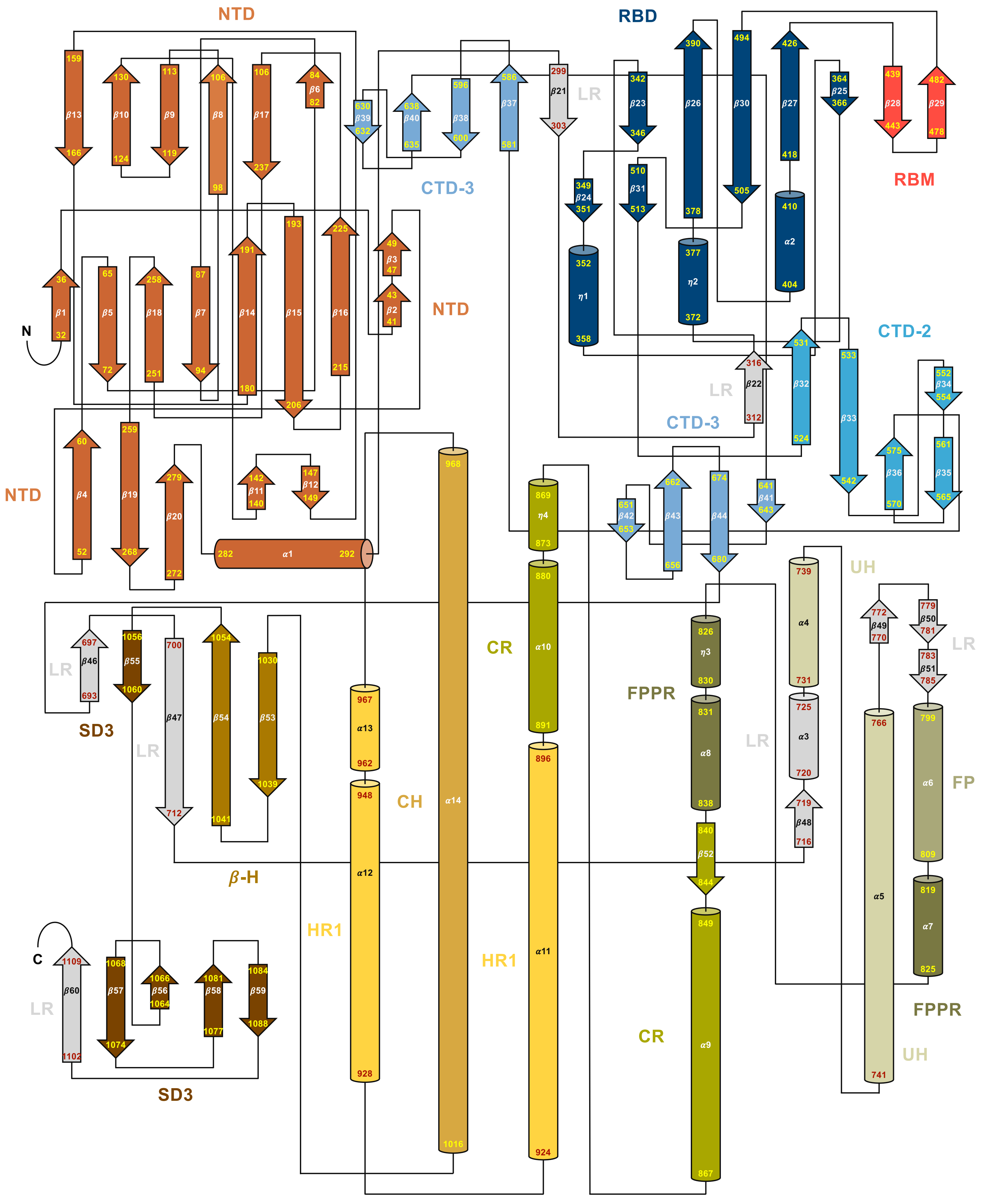

Fig. S3. Structural topology of bat SL-CoV WIV1 S. Structural organization of bat SL-CoV WIV1 S ectodomain (aa 20-1194), with α-helices (α1 - α14), 3_{10}-helices (η1 - η4) and β-strands (β1 - β60) depicted as cylinders and arrows, respectively; amino acid positions defining boundaries of secondary structures are indicated at the beginning and the end of cylinders and arrows, whereas the different domains within WIV1 S are denoted by the following colors: N-terminal domain (NTD), medium vermilion; receptor binding domain (RBD), dark midnight blue; receptor binding motif (RBM), deep carmine pink; C-terminal domain 2 (CTD-2), moonstone; C-terminal domain 3 (CTD-3), blue-gray; upstream helix (UH), dark vanilla; fusion peptide (FP), moss green; fusion peptide proximal region (FPPR), mustard green; connecting region (CR), dark yellow; heptad repeat 1 (HR1), sunglow; central helix (CH), satin sheen gold; β-hairpin (β-H), gamboge orange; subdomain 3 (SD3), Philippine bronze; α-helix and β-strand linker regions (LR), Chinese silver.

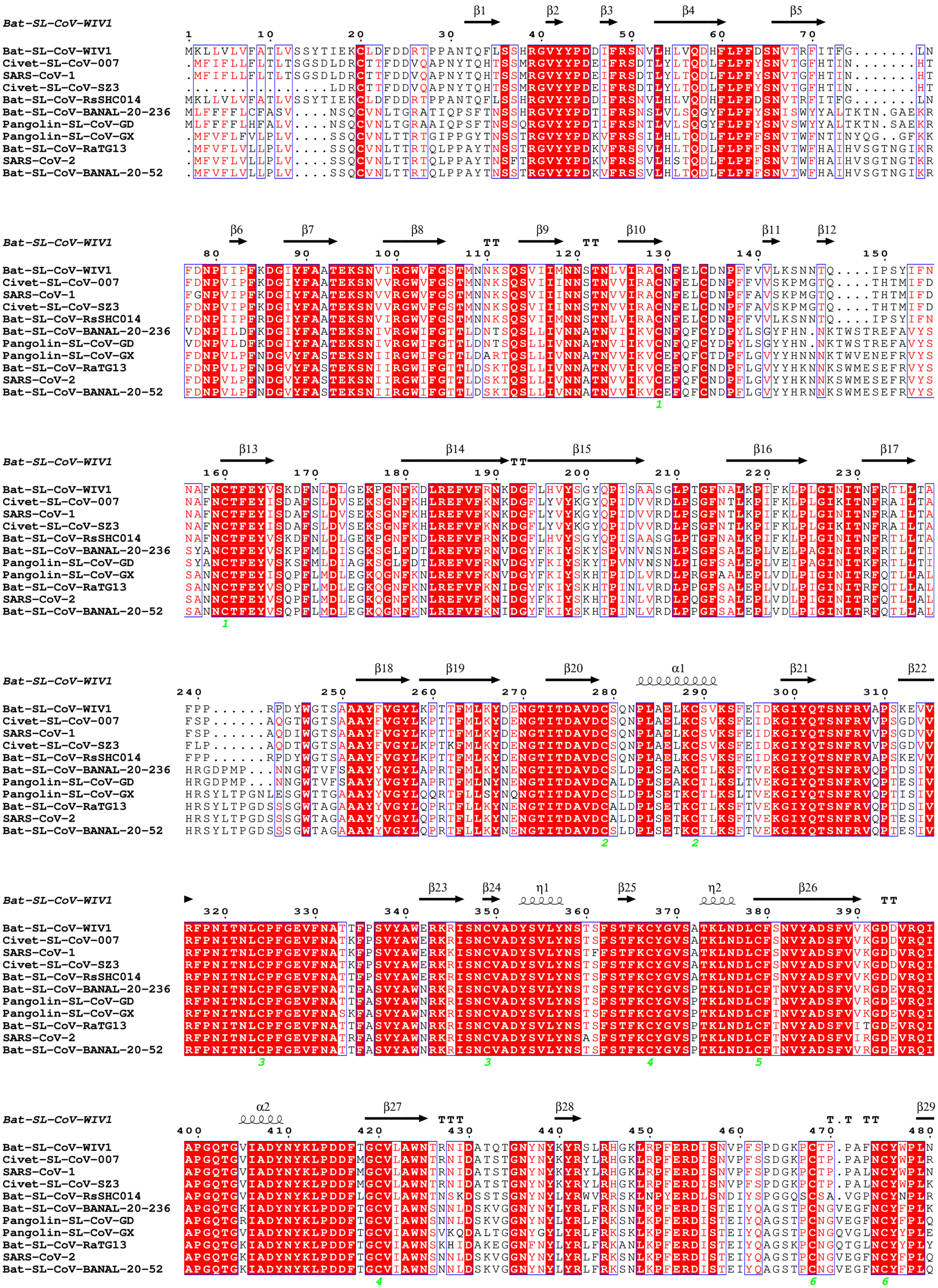

Fig. S4. Multiple sequence alignment of S glycoprotein amino acid sequences from SL-CoVs based on consensus with topology of bat SL-CoV WIV1 S structure (this work). Alignment (aa 1-1256, WIV1 numbering) was performed by using S reference sequences within the family *Coronaviridae*, genus *Betacoronavirus*, subgenus *Sarbecovirus*, retrieved from the NCBI Protein database with the following accession codes: bat SL-CoV WIV1, AGZ48831.1; civet SL-CoV 007, AAU04646.1; SARS-CoV-1, YP_009825051.1; Civet SL-CoV SZ3, AY304486.1; bat SL-CoV RsSHC014, AGZ48806.1; bat SL-CoV BANAL-236, UAY13253.1; pangolin SL-CoV GD, QIG559451.1; pangolin SL-CoV GX, QIA48614.1; bat SL-CoV RaTG13, QHR63300.2; SARS-CoV-2, YP_009724390; bat SL-CoV BANAL-20-52, UAY13217.1. The secondary structure topology scheme from WIV1 S atomic model (this work) is used as consensus and displayed above the aligned sequences.

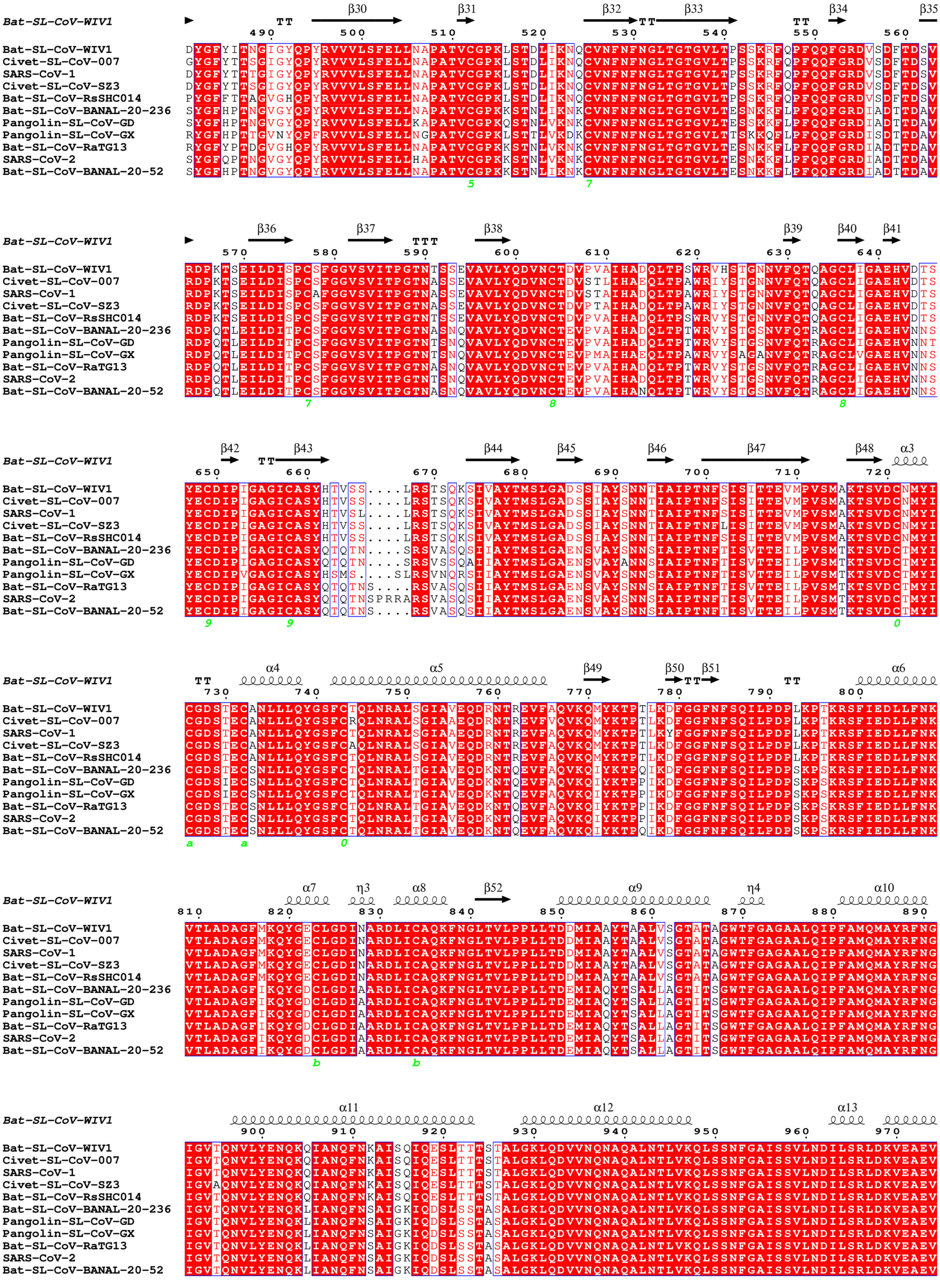

Fig. S4 (continued). Multiple sequence alignment of S glycoprotein amino acid sequences from SL-CoVs based on consensus with topology of bat SL-CoV WIV1 S structure (this work).

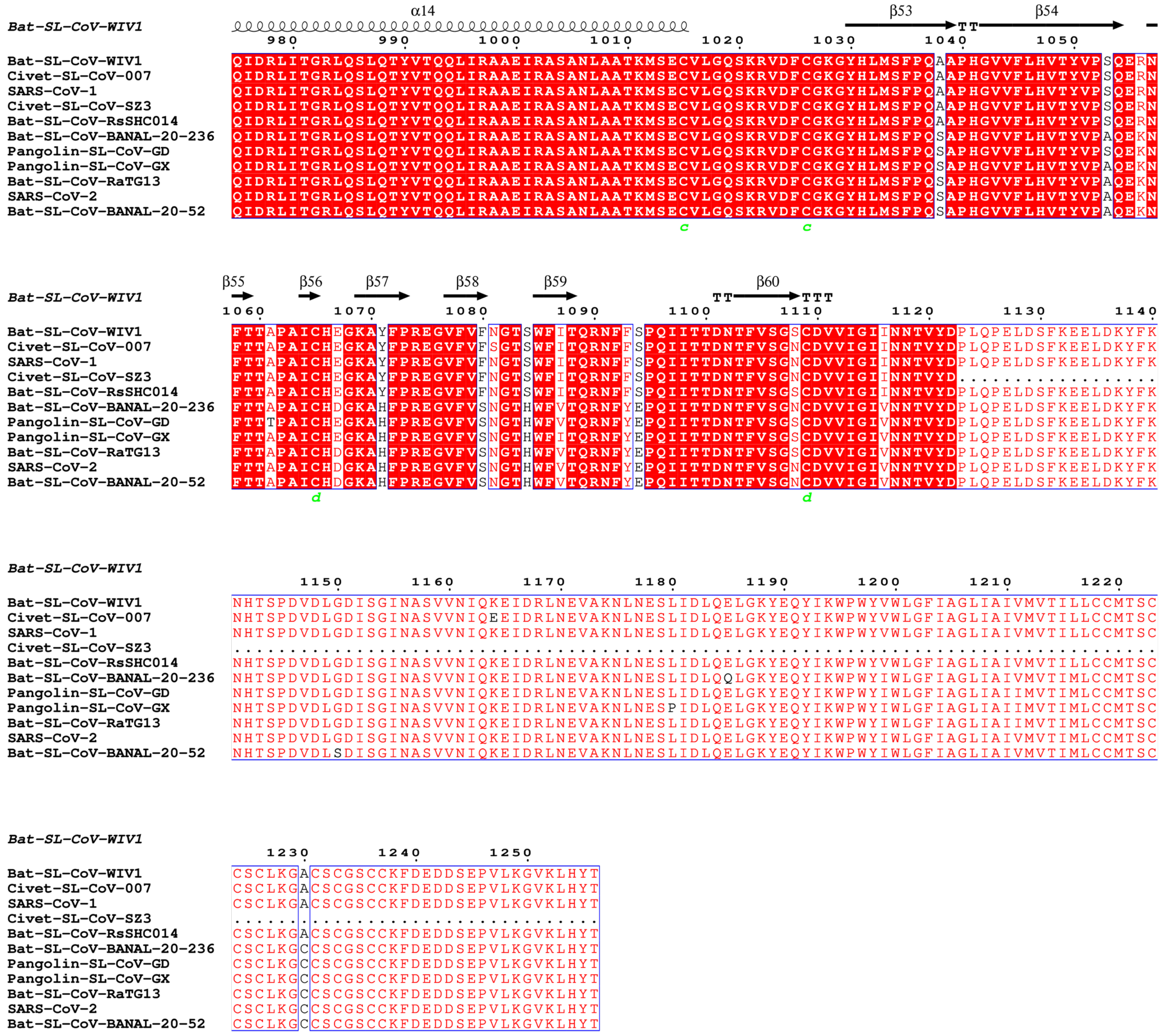

Fig. S4 (continued). Multiple sequence alignment of S glycoprotein amino acid sequences from SL-CoVs based on consensus with topology of bat SL-CoV WIV1 S structure (this work).

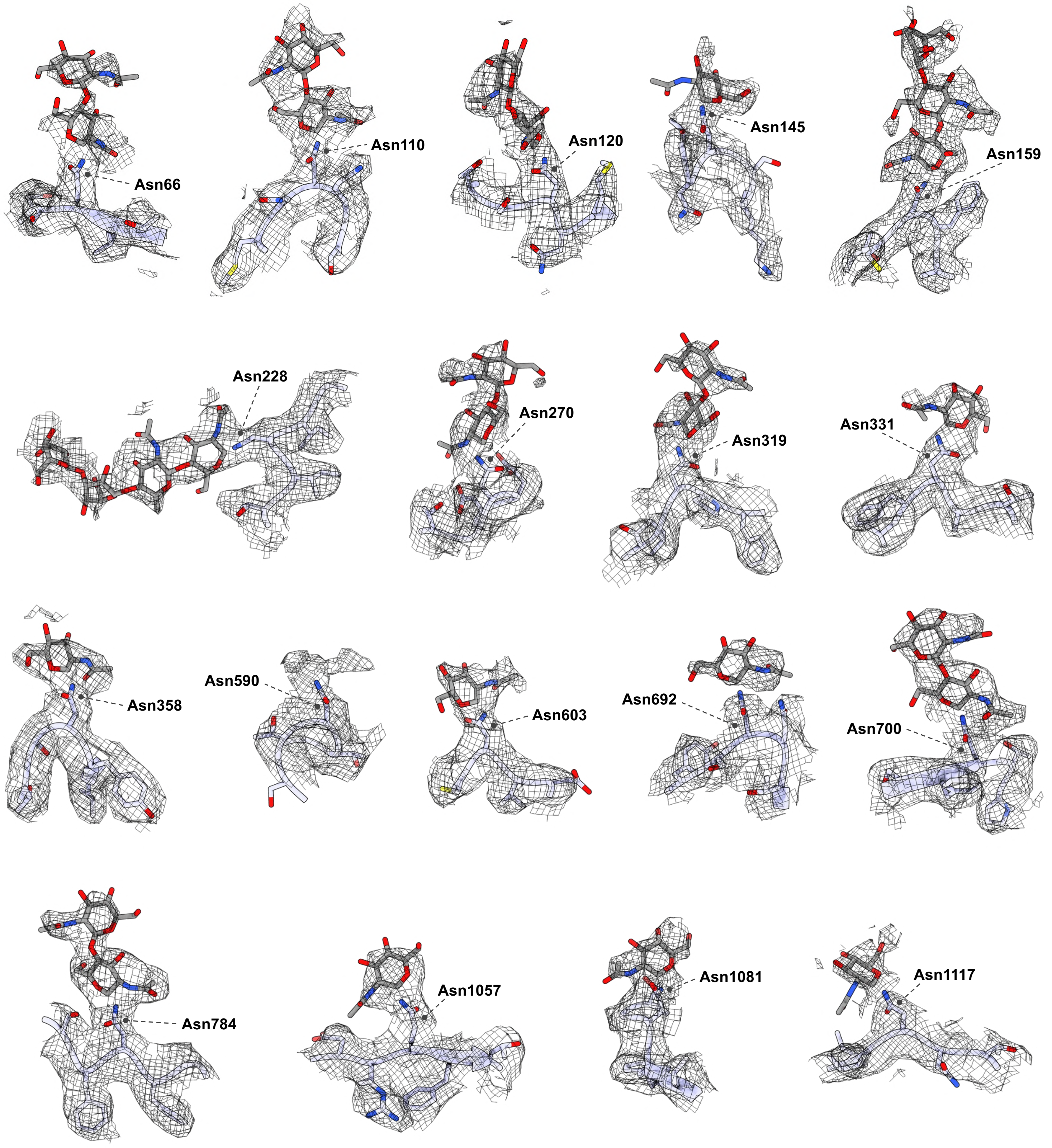

Fig. S5. Glycosylation of locked-like bat SL-CoV WIV1 S. Representative densities and fitted built atomic models, shown as gray mesh and colored ribbon cartoons, respectively, of glycans (Spanish gray, by heteroatoms) N-linked to WIV1 S (this work, ceil violet), with Asparagine and adjacent amino acid side chains represented as sticks and colored by heteroatoms; atomic model of glycan linked to Asn590 is omitted due to ambiguity of orientation within the relative density.

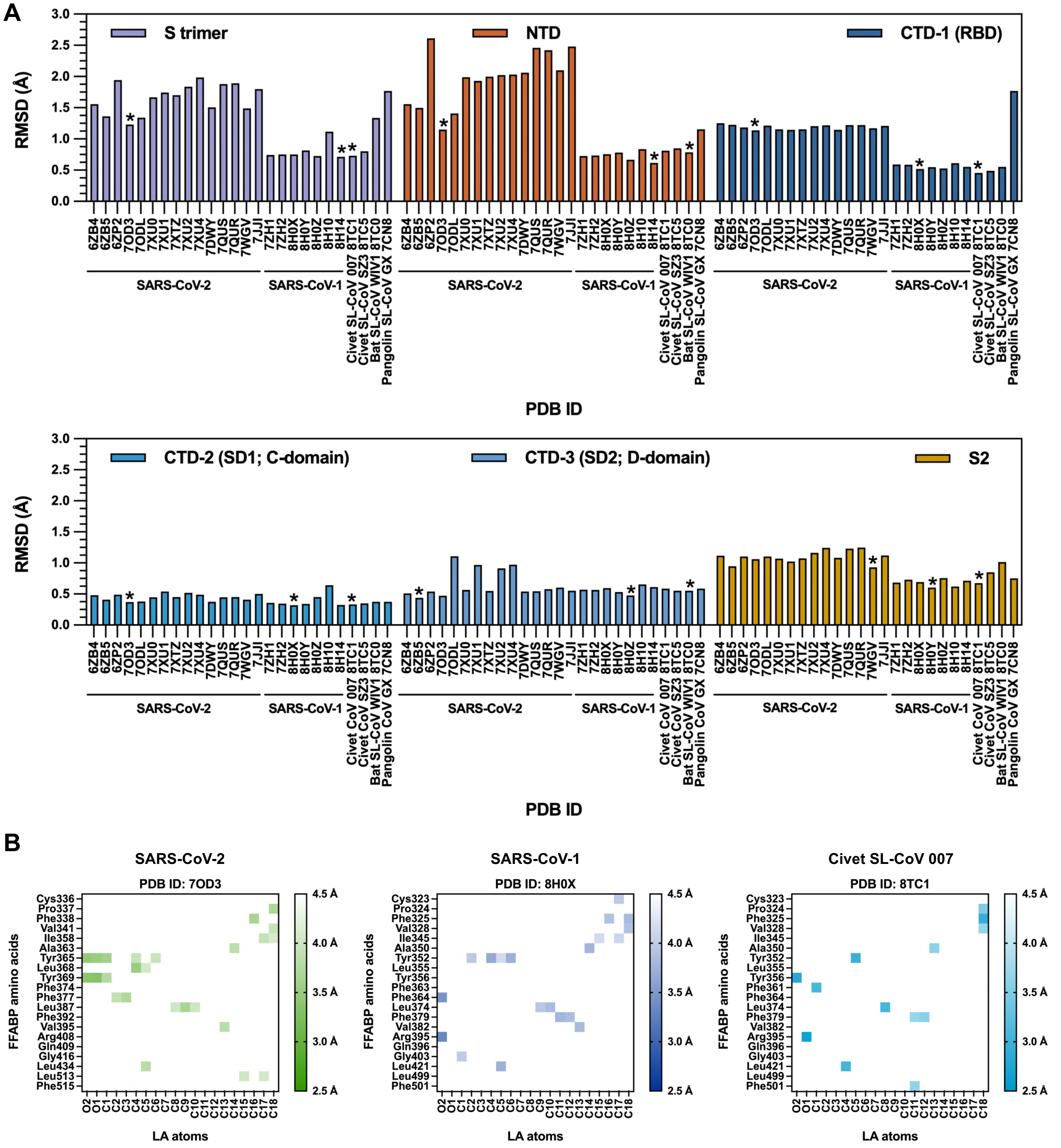

Fig. S6. Similarity of bat SL-CoV WIV1 S with ortholog structures in the prefusion state and locked-like conformations. (*A*) Structural alignment between the bat SL-CoV WIV1 S structure (this work) and those in the PDB of representative orthologs from other SL-CoVs, whose RBDs are complexed to LA; RMSD values from structural alignment are plotted differentially, either for the entire S homo-trimer as well as, within the best-paired monomeric chain, for the isolated NTD, RBD, CTD-2, CTD-3 and S2 domain; most similar structures to WIV1 S (i.e., those displaying the lowest RMSD values) among SARS-CoV-2, SARS-CoV-1 and other SL-CoVs in each structural alignment are highlighted by an asterisk. (*B*) Residue contact heatmaps of FFABP amino acid residues interacting with LA heavy atoms (within 4.5 Å distance cutoff) in three representative S structures from SARS-CoV-2 (PDB ID: 7OD3, left panel), SARS-CoV-1 (PDB ID: 8H0X, middle panel) and civet SL-CoV 007 (PDB ID: 8TC1, right panel), which top-ranked in the structural alignment with WIV1 S for the isolated RBD; interactions are plotted as squares colored in different shades of slimy green, dark powder blue and rich electric blue, respectively, where color intensity increases inversely with the distance.

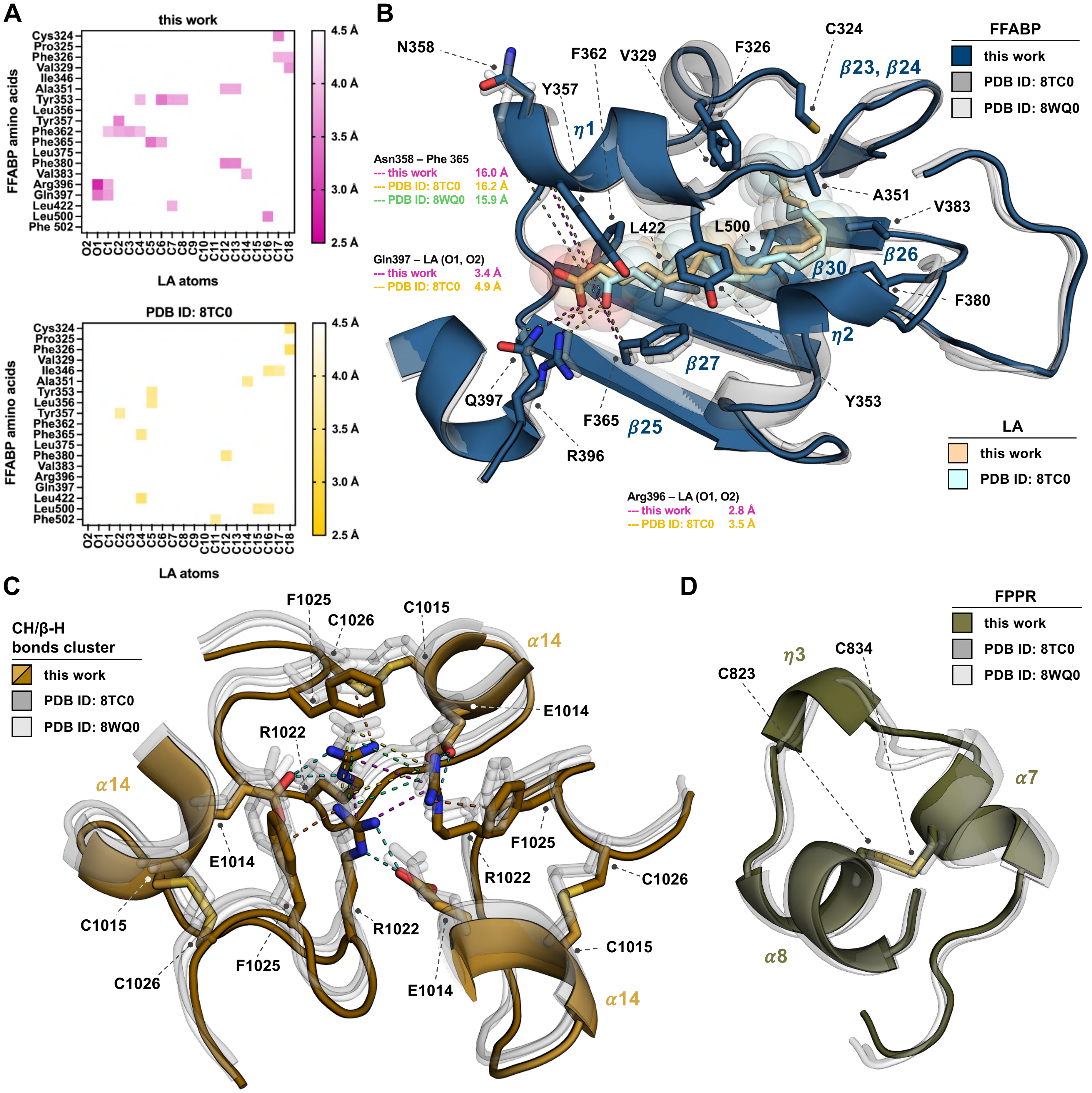

Fig. S7. Molecular signatures of locked-1 conformation in bat SL-CoV WIV1 S structure. (*A*) Residue contact heatmaps of the interactions between S RBD-FFABP amino acids and LA heavy atoms, existing within 4.5 Å distance cutoff in the WIV1 S structure presented in this work (upper panel) as compared to the one in PDB ID: 8TC0 (lower panel); interactions are plotted as squares colored in different shades of medium violet-red and tangerine yellow, respectively, where color intensity increases inversely with the distance. (*B*) Structural alignment of superposed RBD-FFABP from the LA-bound WIV1 S structures presented in this work (dark midnight blue), and in PDB ID: 8TC0 (Spanish gray), and the LA-undetected one in PDB ID: 8WQ0 (Chinese silver), shown in ribbon representation; LA molecules from this work (peach orange) and PDB ID: 8TC0 (water) are both shown as sticks and transparent atom spheres representation. (*C*) Structural alignment of superposed triangular bonds cluster between CH and β-H from WIV1 S structures described in this work (satin sheen gold and gamboge orange for CH and β-H, respectively), in PDB ID: 8TC0 (Spanish gray) and PDB ID: 8WQ0 (Chinese silver). (*D*) Structural alignment of superposed three-helix highly ordered FPPR from WIV1 S structure presented in this work (mustard green) and two-helix partially ordered ones in PDB ID: 8TC0 (Spanish gray) and PDB ID: 8WQ0 (Chinese silver). For all superpositions, orthologs to the structure presented in this work are shown as transparent ribbons, side chains of interacting residues are shown as sticks representation and colored by heteroatoms, secondary structure elements are indicated, and distances between representative residues are shown as dashed lines (medium violet-red, Chinese gold and Mantis, respectively).

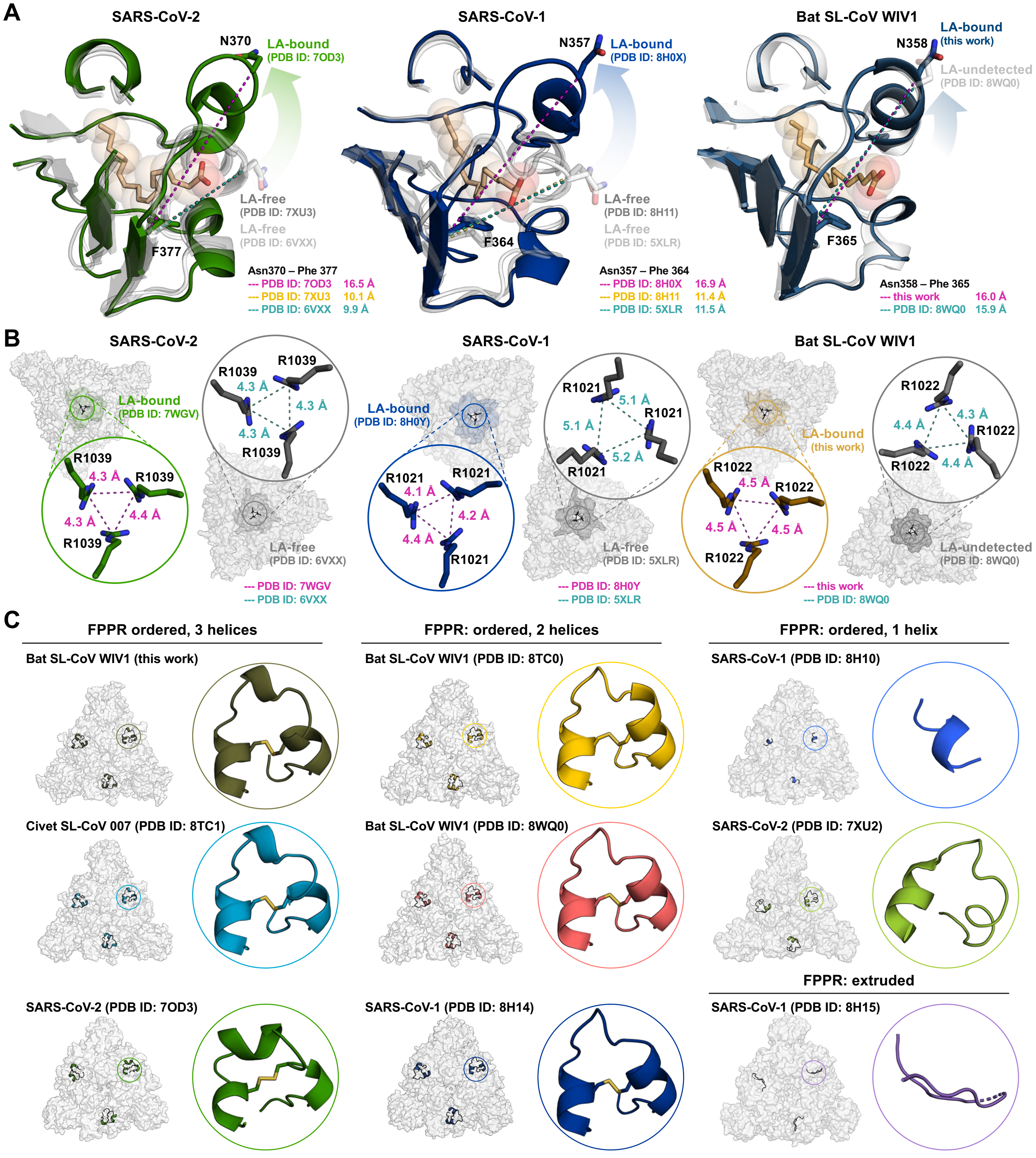

Fig. S8. Structural analysis of bat SL-CoV WIV1 S locked-1 conformation hallmarks. (*A*) Structural alignment of LA-bound RBD-FFABP ribbon representation from SARS-CoV-2 (left panel, PDB ID: 7OD3, slimy green) SARS-CoV-1 (middle panel, PDB ID: 8H0X, dark powder blue) and bat SL-CoV WIV1 S (right panel, this work, dark midnight blue) superposed with representative LA-free orthologs (PDB IDs: 7WGV, 6VXX; 8H0Y, 5XLR) and WIV1 S without LA annotation (PDB ID: 8WQ0), showing the D_pocket increase due to LA accommodation; for all superpositions, LA-free structures are shown as transparent ribbon representation (granite gray and argent, respectively) and distances as dashed lines (medium violet-red, Chinese gold and Zomp, respectively); LA molecules (peach orange) are shown as sticks and transparent atom spheres representation, and side chains as sticks representation colored by heteroatoms. (*B*) Bottom views of LA-bound and LA-free SARS-CoV-2 (left panel, PDB IDs: 7WGV and 6VXX, respectively), SARS-CoV-1 (middle panel, PDB IDs: 8H0Y and 5XLR, respectively) and WIV1 S (right panel, this work and PDB ID: 8WQ0, respectively) shown as transparent isosurface representation (argent) with highlighted CH/β-H (granite gray, slimy green, dark powder blue, satin sheen gold, respectively); triangular Arginine clusters are shown as sticks representation in zoomed insets, with side chains colored by heteroatoms and H-bond distances as dashed lines (medium violet-red, Chinese gold and Zomp color, for LA-bound and LA-free structures, respectively). (*C*) Top views of SL-CoV S shown as transparent isosurface (argent) with FPPR in different conformations highlighted as ribbon representation (also in zoomed insets) from SARS-CoV-2 (PDB IDs: 7OD3, slimy green; 7XU2, yellow-green), SARS-CoV-1 (PDB IDs: 8H10, ultramarine blue; 8H14, dark powder blue; 8H15, amethyst), civet SL-CoV 007 (PDB ID: 8TC1, rich electric blue) and WIV1 S (this work, dark midnight blue; PDB IDs: 8TC0, tangerine yellow; 8WQ0, pastel red).

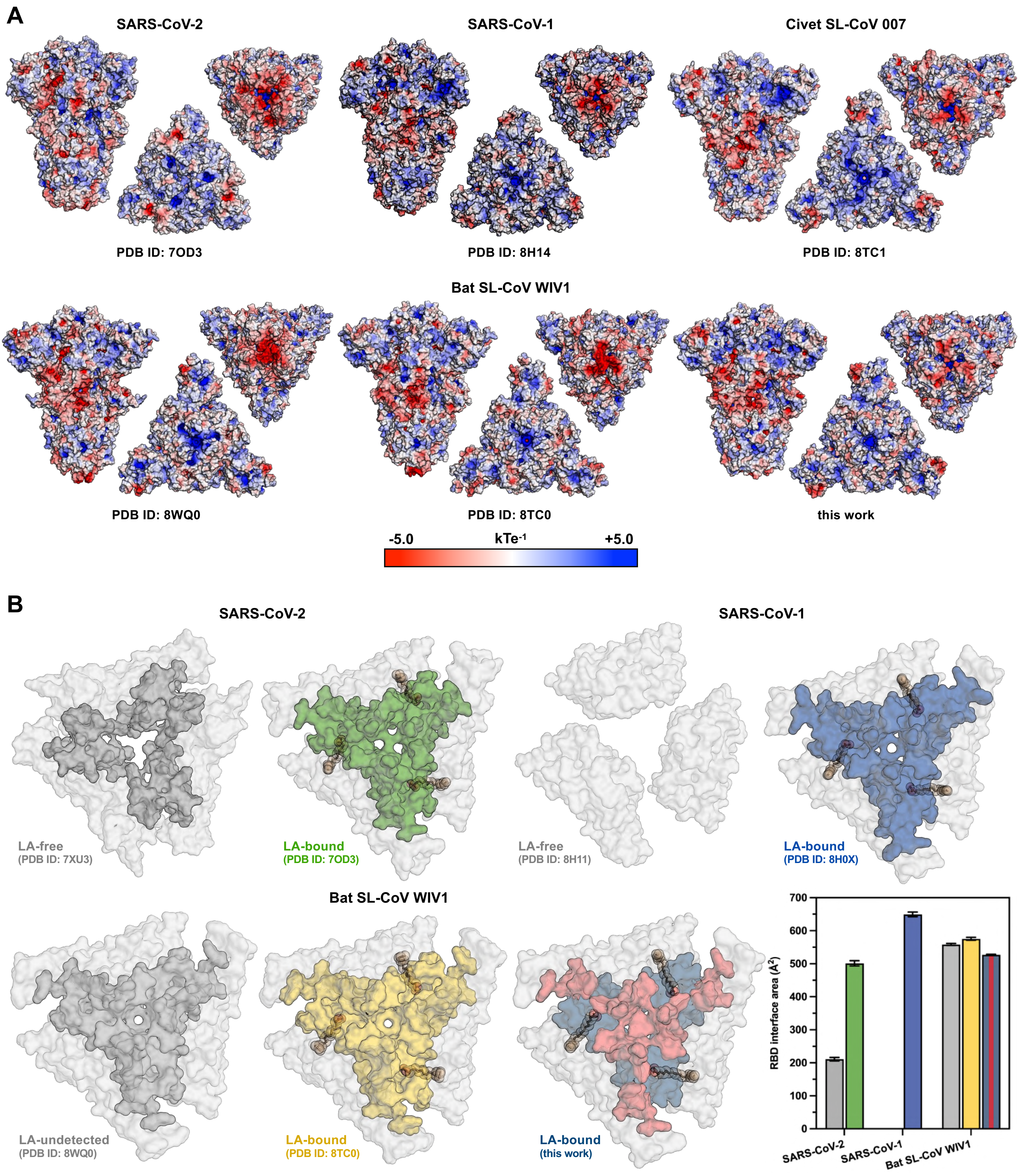

Fig. S9. Comparative structural analysis of bat SL-CoV WIV1 S molecular surface properties and inter-RBD interactions. (*A*) Electrostatic surface potential calculation showing different surface charge patterns in LA-bound SARS-CoV-2 (PDB ID: 7OD3), SARS-CoV-1 (PDB ID: 8H14) and civet SL-CoV 007 (PDB ID: 8TC1), LA-undetected bat SL-CoV WIV1 (PDB ID: 8WQ0) and LA-bound WIV1 S (PDB ID: 8TC0), as compared to LA-bound WIV1 S (this work), all displayed as isosurface representation of side, top and bottom views; red, white and blue areas represent regions with negative, neutral, and positive electrostatic surface potential, respectively (scale bar, from -5.0 to + 5.0 kT e^{-1}). (*B*) Top views of LA-free and LA-bound S RBDs from SARS-CoV-2 (PDB IDs: 7XU3 and 7OD3, respectively), SARS-CoV-1 (PDB IDs: 8H11 and 8H0X, respectively), LA-undetected and LA-bound WIV1 S (PDB IDs: 8WQ0 and 8TC0, respectively) showing difference in the interacting surface between RBDs as compared to LA-bound WIV1 S RBD (this work); RBDs and RBD-interacting regions are shown as transparent (argent) and colored (granite gray for LA-free RBDs, slimy green, dark powder blue, tangerine yellow and dark midnight blue for LA-bound RBDs) isosurface, respectively; RBMs in LA-bound WIV1 S (this work) are colored in deep carmine pink; LA molecules (peach orange) are shown as sticks and transparent atom spheres representation, colored by heteroatoms; a quantitative analysis of the RBD interface areas between LA-free and LA-bound SARS-CoV-2, SARS-CoV-1, LA-undetected and LA-bound WIV1 S RBDs is shown as histogram (bottom right) with error bars as mean and standard deviations of three-chain values averages.

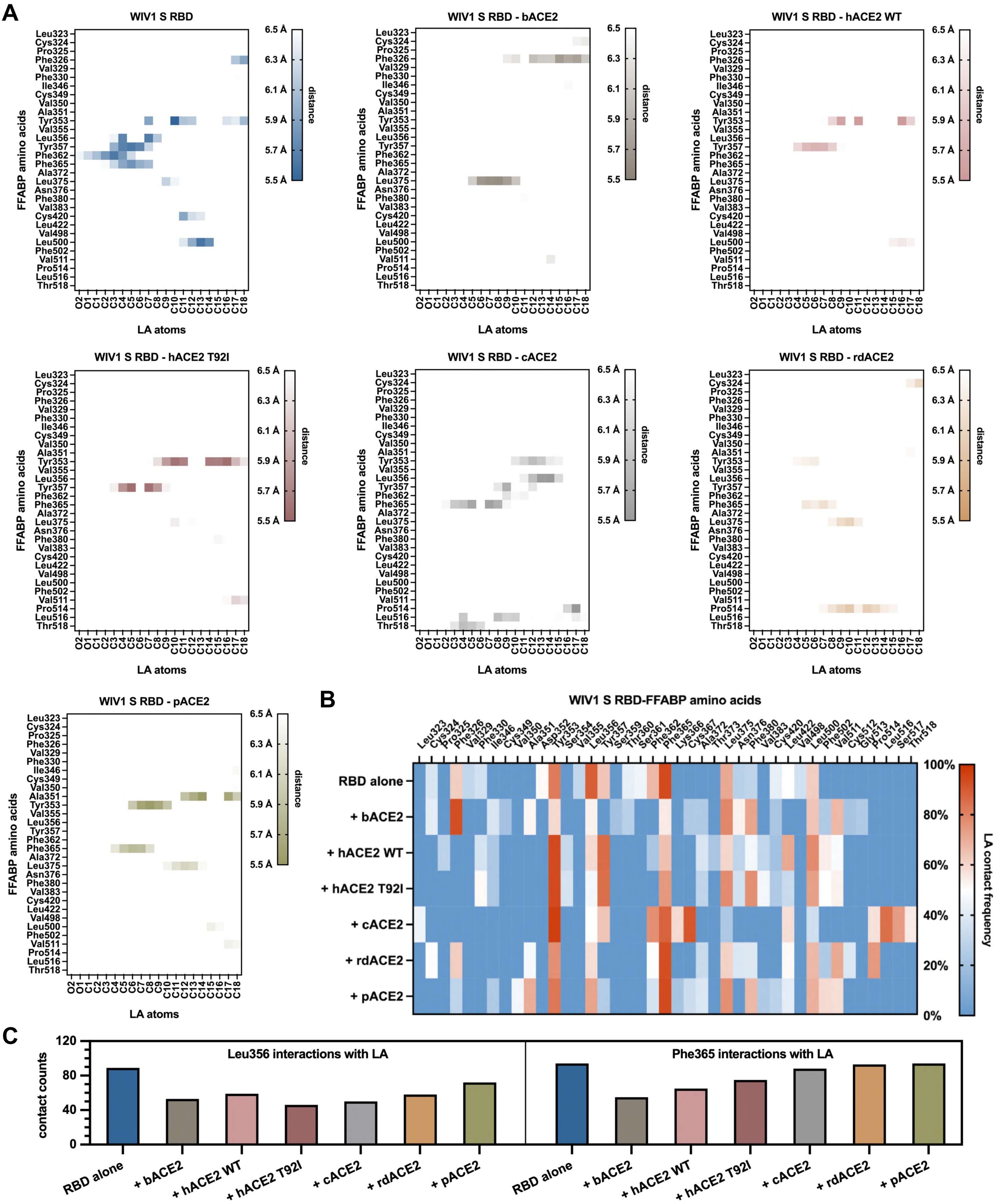

Fig. S10. Comparative analysis of bat SL-CoV WIV1 S RBD-LA interactions in RBD-ACE2 complexes. (*A*) Residue contact heatmaps of WIV1 S RBD-FFABP amino acid residues (and neighboring ones in Domain C) interacting with LA heavy atoms (between 5.5 Å and 6.5 Å distance) after MD simulation, calculated for the RBD alone and for complex with bACE2, hACE2 WT, hACE2-T92I mutant, cACE2, rdACE2 and pACE2; interactions are plotted as squares colored in different shades of colors (dark midnight blue, rocket metallic, Tuscany pink, copper rose, Spanish gray, brown yellow and moss green, respectively), where color intensity increases inversely with the distance. (*B*) Contact frequency heatmap of WIV1 S RBD-FFABP amino acid residues (and neighboring ones in Domain C) with LA heavy atoms during MD simulation, in the RBD alone and in complex with bACE2, hACE2 WT, hACE2-T92I mutant, cACE2, rdACE2 and pACE2; color intensity varies according to contact frequency percentage over simulation time (0%-100% over Blue-gray, white and Mahogany gradient, respectively). (*C*) Counts of interactions established by WIV1 S RBD-FFABP Leu356 and Phe365 with LA heavy atoms during MD simulation, calculated (within 4.0 Å distance) for the RBD alone and in complex with bACE2, hACE2 WT, hACE2-T92I mutant, cACE2, rdACE2 and pACE2. Analyses are the average of last 100 ns of total (500 ns) MD simulation time.

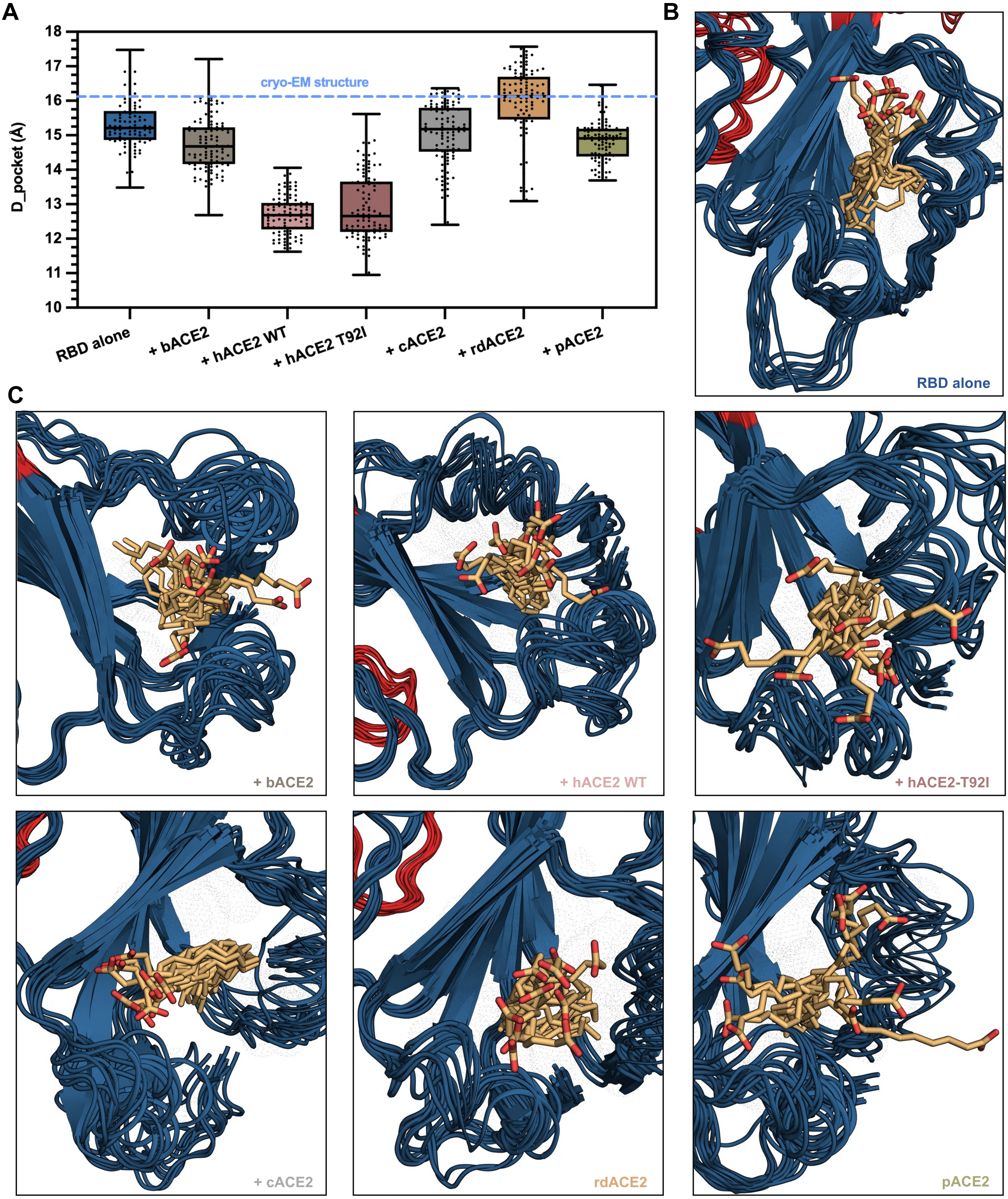

Fig. S11. MD of the reduction of D_pocket distance in bat SL-CoV WIV1 S RBD-FFABP during transition from locked-1 to unlocked conformation. (*A*) Change in the FFABP D_pocket (distance between Asn358 and Phe365) during the last 100 ns of the total (500 ns) MD simulation, calculated for the isolated WIV1 S RBD and for its complex with bACE2, hACE WT, hACE2 T92I mutant, cACE2, rdACE2 and pACE2; continuous line in the middle of each box represents the median value, with top and bottom boundaries of the box corresponding to the upper and lower quartiles, respectively, and error bar referring to the minimum and maximum values of the distribution (also shown as single experimental points); color code for RBD and ACE2 from different hosts is the same as in Figure 2 of main text. Ribbon representation of the dynamics of structural changes during 500 ns MD simulation in (*B*) the isolated WIV1 S RBD-FFABP (dark midnight blue, with RBM highlighted in deep carmine pink) and (*C*) in its complex with bACE2 (upper left panel) hACE WT (upper middle panel) and T92I mutant (upper right panel), cACE2 (lower left panel), rdACE2 (lower middle panel) and pACE2 (lower right panel); LA (peach orange) is shown as stick representation and colored by heteroatoms.

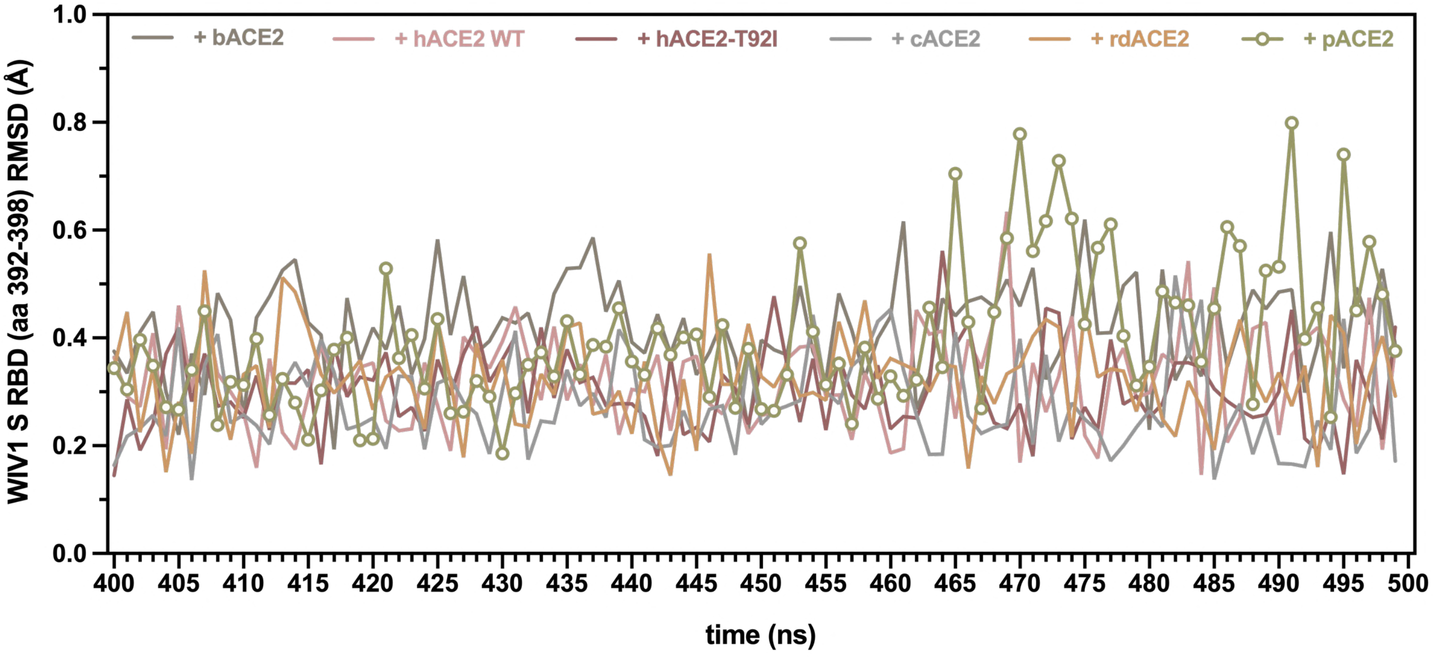

Fig. S12. MD simulation of the aa 392-398 dynamics of bat SL-CoV WIV1 S RBD with ACE2 receptors from different mammalian hosts. RMSD of 100 atomic models from the last 100 ns of MD simulation, relative to the first structure in the set; the RMSD profile for the complexes with the bACE2, hACE2 WT and T92I mutant, cACE2 and rdACE2 are represented by continuous lines, whereas the one for the complex with pACE2 is represented by continuous-line connected dots. Color code for ACE2 from different hosts is the same as in Figure S9.

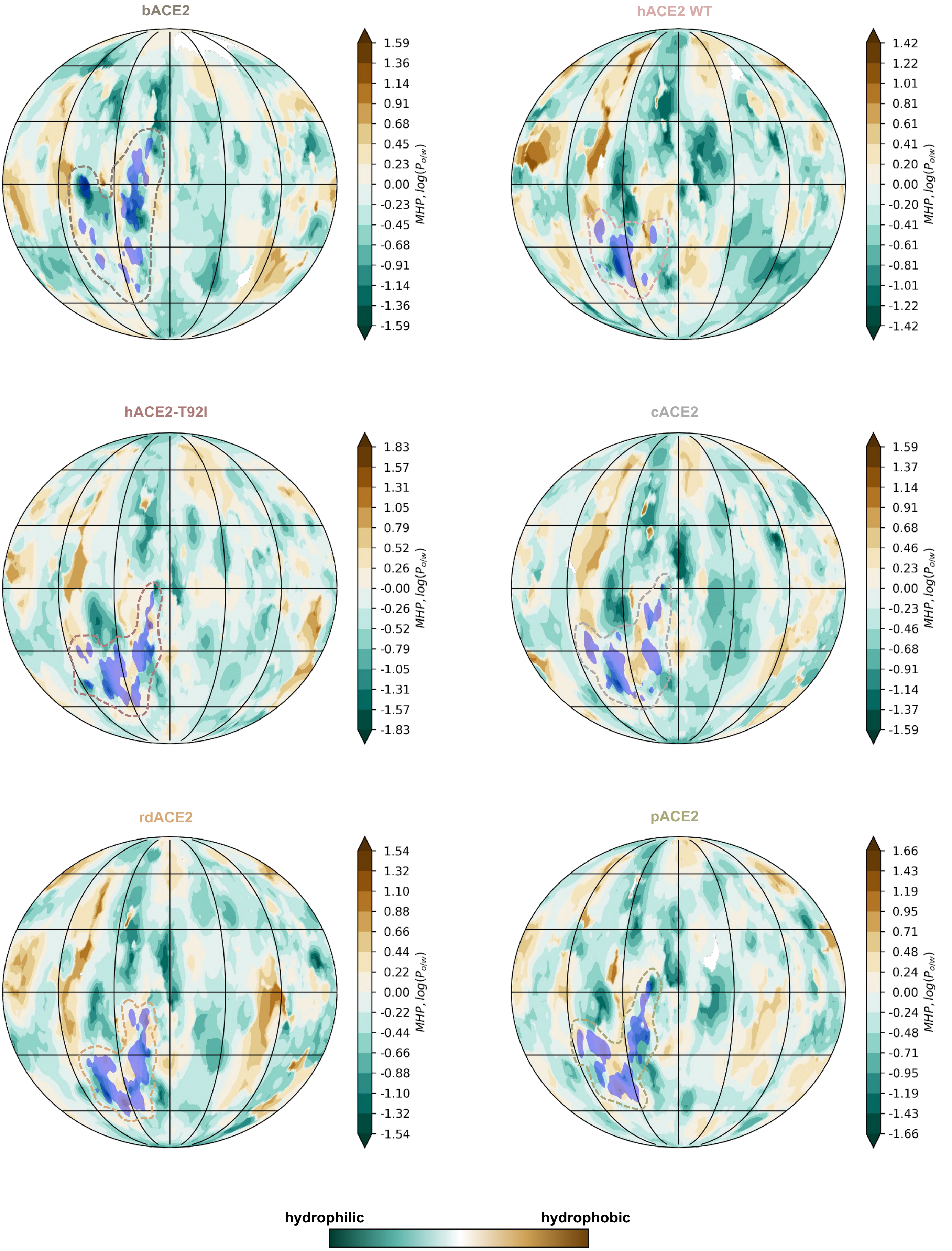

Fig. S13. Footprint of ACE2 from different mammalian hosts on the surface of bat SL-CoV WIV1 S RBD. PST analysis of the contact 2D spherical contact resulting from the complex formation between WIV1 S RBD and bACE2 (upper left), hACE2 WT (upper middle), hACE2-T92I (upper right), cACE2 (lower left), rdACE2 (lower middle) and pACE2 (lower right); surface of WIV1 S RBD is colored by a (Sacramento State Green to Philippine Bronze) color gradient according to molecular hydrophobicity potential (MHP) values, whereas footprint of ACE2 contact within 4.0 Å cutoff is colored in blue and highlighted by a dashed line contour. Color code for ACE2 from different hosts is the same as in Figure S9.

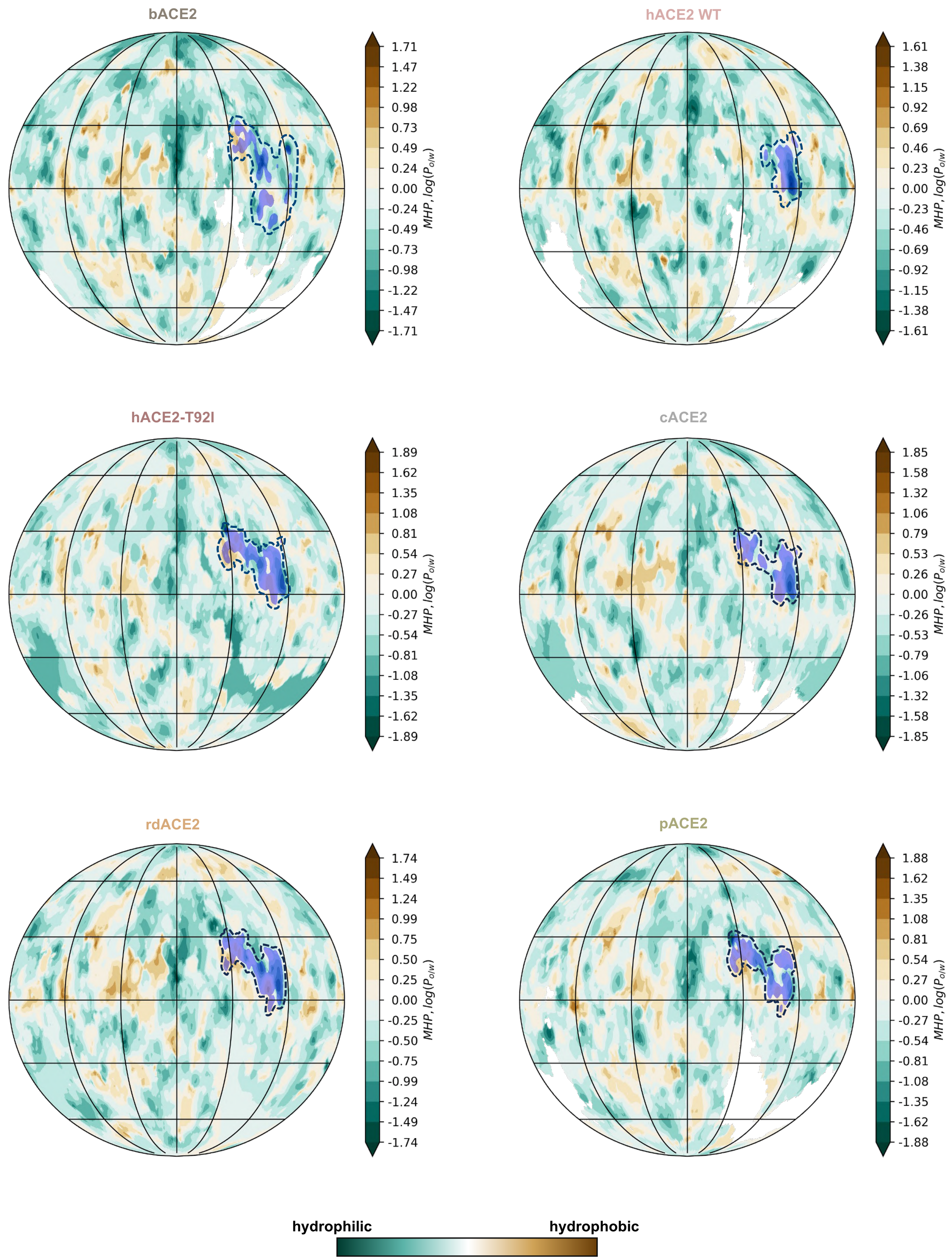

Fig. S14. Footprint of bat SL-CoV WIV1 S RBD on the surface of ACE2 from different mammalian hosts. PST analysis of the contact 2D spherical map resulting from the complex formation between WIV1 S RBD and bACE2 (upper left), hACE2 WT (upper middle), hACE2-T92I (upper right), cACE2 (lower left), rdACE2 (lower middle) and pACE2 (lower right); surface of ACE2 receptor is colored by a (Sacramento State Green to Philippine Bronze) color gradient according to molecular hydrophobicity potential (MHP) values, whereas footprint of S RBD contact within 4.0 Å cutoff is colored in blue and highlighted by a dashed line contour. Color code for ACE2 from different hosts is the same as in Figure S9.

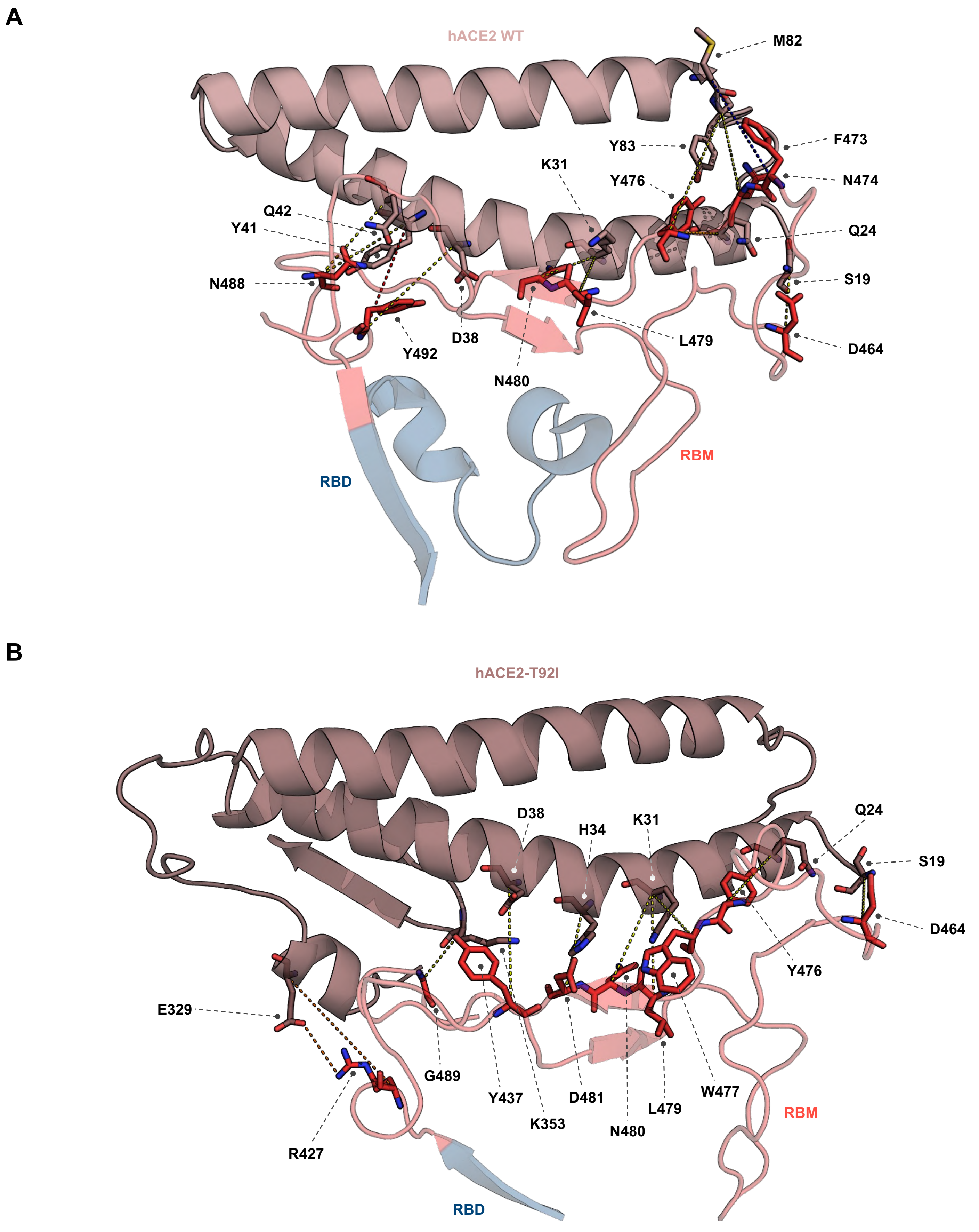

Fig. S15. MD analysis of the average interactions (occurring in more than 60% of the last 100 ns) between bat SL-CoV WIV1 S RBD and hACE2. (*A*) Ribbon representation of the interactions between WIV1 S RBD (dark midnight blue, RBM highlighted in deep carmine pink) and hACE2 WT (Tuscany pink), where yellow lines represent H-bonds, blue lines indicate hydrophobic interactions, red lines denote π-π interactions, and orange lines represent salt bridges; side chains of interacting aa are indicated, shown as sticks and colored by heteroatoms. (*B*) Ribbon representation of the interactions between WIV1 S RBD and hACE2-T92I mutant (copper rose), with the same color annotation of interactions and interacting residues as in panel A.

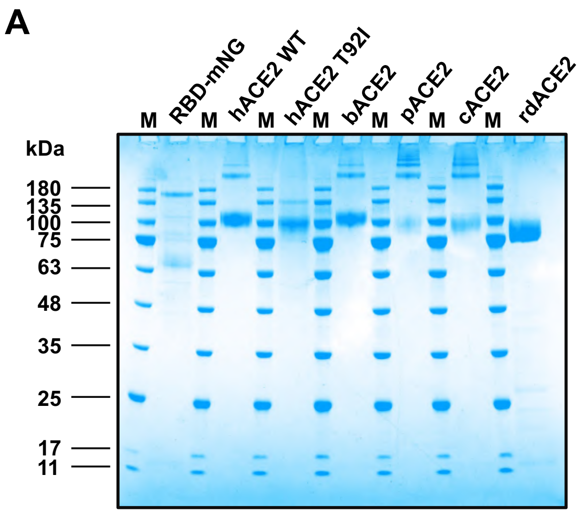

B

construct	theoretical MW (Da)
WIV1 S RBD-mNG	53,769.43
bACE2	72,681.29
hACE2 WT	71,553.55
hACE2 T92I	72,283.14
cACE2	72,351.55
rdACE2	72,499.71
pACE2	72,413.81

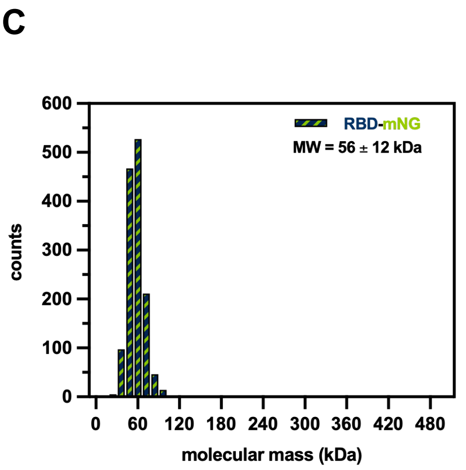

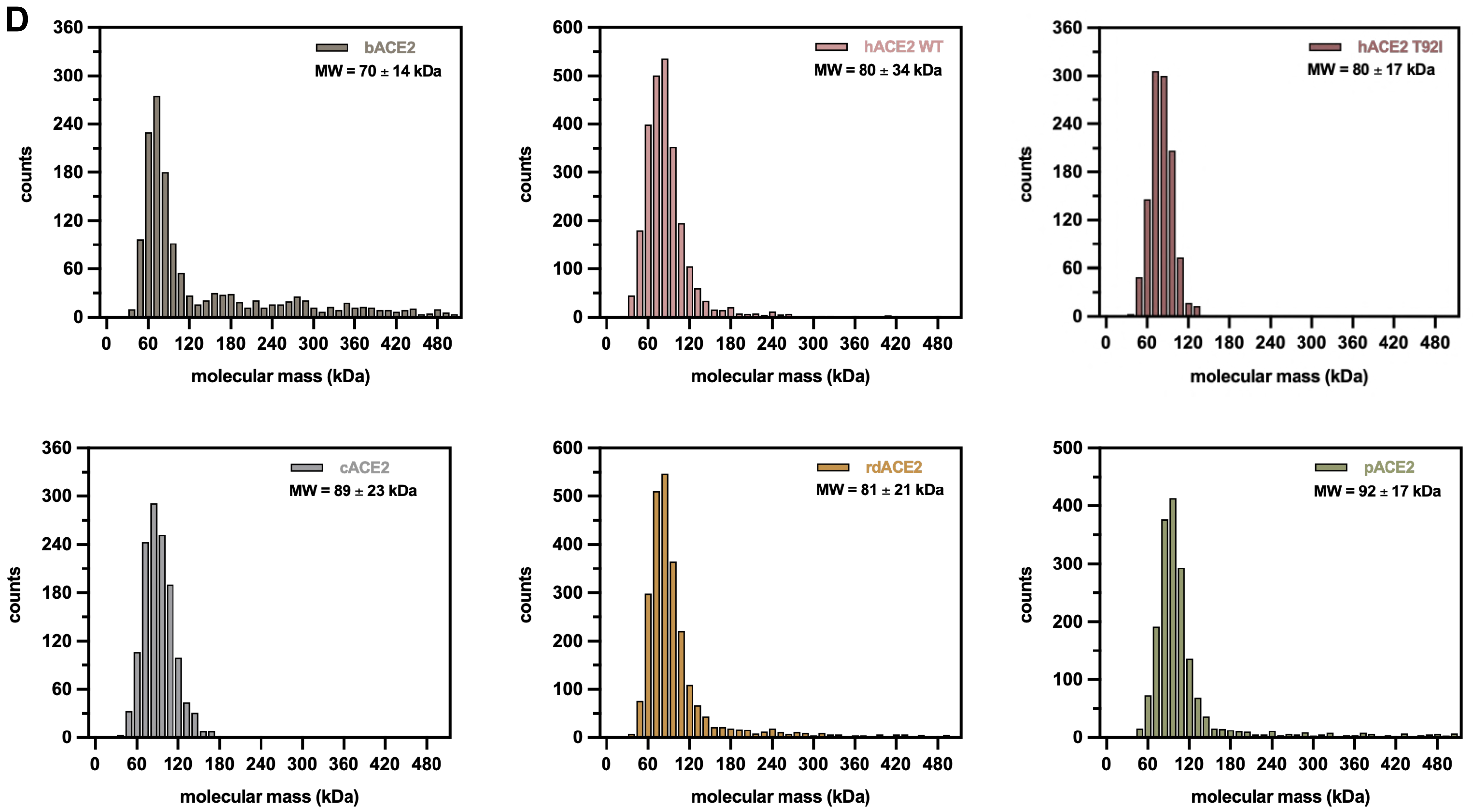

Fig. S16. Purification and MP analysis of recombinant bat SL-CoV WIV1 S RBD and mammalian ACE2. (*A*) SDS-PAGE analysis of HEK293F-expressed and purified recombinant WIV1 S RBD-mNG and ACE2 from human (WT and T92I mutant), bat, pangolin, civet and raccoon dog hosts (M, molecular weight marker). (*B*) Summary table of the theoretical MW expected for monomeric (non-glycosylated) recombinant WIV1 S RBD-mNG and ACE2s from different hosts. MP histograms of molecular mass distribution showing the in-solution quaternary structure of purified recombinant (*C*) WIV1 S RBD-mNG and (*D*) bACE2 (upper left panel), hACE2 WT and T92I (upper middle and right panels, respectively), cACE2 (lower left panel), rdACE2 (middle left panel) and pACE2 (lower right panel); mean and standard deviation of MW values relative to the histogram peak are indicated. RBD and mNG are colored in dark midnight blue and sheen green, respectively, whereas color code for ACE2 from different hosts is the same as in Figure S9.

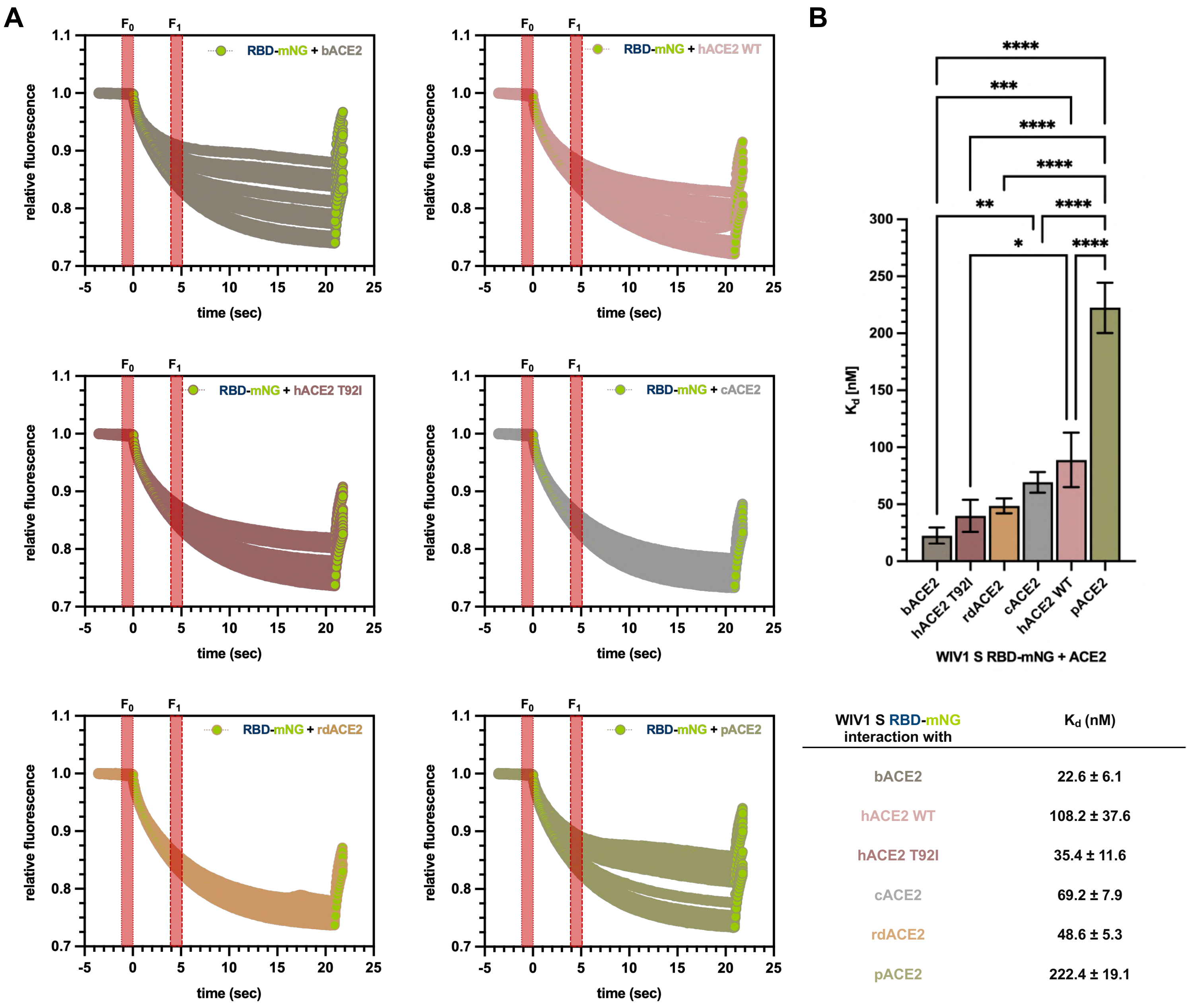

WIV1 S RBD-mNG interaction with	K_d (nM)
bACE2	22.6 ± 6.1
hACE2 WT	108.2 ± 37.6
hACE2 T92I	35.4 ± 11.6
cACE2	69.2 ± 7.9
rdACE2	48.6 ± 5.3
pACE2	222.4 ± 19.1

Fig. S17. MST analysis of the interaction between recombinant bat SL-CoV WIV1 S RBD and mammalian ACE2. (*A*) MST traces for the titration of bACE2, (upper left panel), hACE2 WT and T92I mutant (upper left and middle left panels, respectively), cACE2 (middle right panel), rdACE2 (lower left panel) and pACE2 (lower right panel) against WIV1 S RBD-mNG; Cold (F_0) and heated (F_1) state regions for the measurement of the Fnorm (F_1 F_0^{-1} ‰) ratio are indicated as dotted and dashed rectangular areas, respectively. (*B*) Summary histogram (upper panel) and table (lower panel) of the WIV1 S RBD-mNG binding affinity for ACE2 from different mammalian hosts; apparent K_d values are expressed as mean and standard deviation of at least four independent replicates; the number of asterisks is proportional to the level of statistical significance as per one-way ANOVA coupled to Tukey's multiple comparison (P value summary: ∗∗∗∗, < 0.0001; ∗∗∗, < 0.0005; ∗∗, < 0.0065; ∗, < 0.0137). RBD and mNG are labeled in dark midnight blue and sheen green, respectively, whereas color code for ACE2 from different hosts is the same as in Figure S9.

References

1. D. N. Mastronarde, Automated electron microscope tomography using robust prediction of specimen movements. *J Struct Biol* **152**, 36–51 (2005).
2. N. Biyani, *et al.*, Focus: The interface between data collection and data processing in cryo-EM. *J. Struct. Biol.* **198**, 124–133 (2017).
3. S. Q. Zheng, *et al.*, MotionCor2: anisotropic correction of beam-induced motion for improved cryo-electron microscopy. *Nat Methods* **14**, 331–332 (2017).
4. K. Zhang, Gctf: Real-time CTF determination and correction. *J Struct Biol* **193**, 1–12 (2016).
5. A. Punjani, J. L. Rubinstein, D. J. Fleet, M. A. Brubaker, cryoSPARC: algorithms for rapid unsupervised cryo-EM structure determination. *Nat Methods* **14**, 290–296 (2017).
6. T. Bepler, K. Kelley, A. J. Noble, B. Berger, Topaz-Denoise: general deep denoising models for cryoEM and cryoET. *Nat Commun* **11**, 5208 (2020).
7. A. Punjani, H. Zhang, D. J. Fleet, Non-uniform refinement: adaptive regularization improves single-particle cryo-EM reconstruction. *Nat Methods* **17**, 1214–1221 (2020).
8. A. Kucukelbir, F. J. Sigworth, H. D. Tagare, Quantifying the local resolution of cryo-EM density maps. *Nat Methods* **11**, 63–65 (2014).
9. Y. Z. Tan, *et al.*, Addressing preferred specimen orientation in single-particle cryo-EM through tilting. *Nat Methods* **14**, 793–796 (2017).
10. W. Song, M. Gui, X. Wang, Y. Xiang, Cryo-EM structure of the SARS coronavirus spike glycoprotein in complex with its host cell receptor ACE2. *PLoS Pathog* **14**, e1007236 (2018).
11. P. D. Adams, *et al.*, PHENIX: a comprehensive Python-based system for macromolecular structure solution. *Acta Crystallogr D Biol Crystallogr* **66**, 213–221 (2010).
12. E. F. Pettersen, *et al.*, UCSF Chimera--a visualization system for exploratory research and analysis. *J Comput Chem* **25**, 1605–1612 (2004).
13. P. Emsley, B. Lohkamp, W. G. Scott, K. Cowtan, Features and development of Coot. *Acta Crystallogr D Biol Crystallogr* **66**, 486–501 (2010).
14. J. Abramson, *et al.*, Accurate structure prediction of biomolecular interactions with AlphaFold 3. *Nature* **630**, 493–500 (2024).
15. L. Li, *et al.*, Broader-species receptor binding and structural bases of Omicron SARS-CoV-2 to both mouse and palm-civet ACE2s. *Cell Discov* **8**, 65 (2022).
16. L. Wu, *et al.*, Molecular basis of pangolin ACE2 engaged by COVID-19 virus. *Chin Sci Bull* **66**, 73-74 (2021).
17. F. Li, W. Li, M. Farzan, S. C. Harrison, Structure of SARS coronavirus spike receptor-binding domain complexed with receptor. Science 309, 1864–1868 (2005).
18. K. Liu, *et al.*, Cross-species recognition of SARS-CoV-2 to bat ACE2. *Proc Natl Acad Sci U S A* **118**, e2020216118 (2021).
19. F.-C. Hsueh, *et al.*, Structural basis for raccoon dog receptor recognition by SARS-CoV-2. *PLoS Pathog* **20**, e1012204 (2024).
20. K. Suryamohan, et al., Human ACE2 receptor polymorphisms and altered susceptibility to SARS-CoV-2. Commun Biol 4, 475 (2021).
21. A. Waterhouse, *et al.*, SWISS-MODEL: homology modelling of protein structures and complexes. *Nucleic Acids Res* **46**, W296–W303 (2018).
22. E. C. Meng, E. F. Pettersen, G. S. Couch, C. C. Huang, T. E. Ferrin, Tools for integrated sequence-structure analysis with UCSF Chimera. *BMC Bioinformatics* **7**, 339 (2006).

23. P. Mark, L. Nilsson, Structure and dynamics of liquid water with different long-range interaction truncation and temperature control methods in molecular dynamics simulations. *J Comput Chem* **23**, 1211–1219 (2002).
24. M. J. Abraham, *et al.*, GROMACS: High performance molecular simulations through multi-level parallelism from laptops to supercomputers. *SoftwareX* **1**, 19–25 (2015).
25. J. Huang, A. D. MacKerell Jr, CHARMM36 all-atom additive protein force field: validation based on comparison to NMR data. *J Comput Chem* **34**, 2135–2145 (2013).
26. S. Jo, *et al.*, CHARMM-GUI 10 years for biomolecular modeling and simulation. *J Comput Chem* **38**, 1114–1124 (2017).
27. B. Hess, H. Bekker, H. J. Berendsen, J. G. Fraaije, Lincs: a linear constraint solver for molecular simulations. *J Comput Chem* **18**, 1463–1472 (1997).
28. M. S. Valdés-Tresanco, M. E. Valdés-Tresanco, P. A. Valiente, E. Moreno, gmx_MMPBSA: A New Tool to Perform End-State Free Energy Calculations with GROMACS. *J Chem Theory Comput* **17**, 6281–6291 (2021).
29. S. Yuan, *et al.*, Conformational Dynamics of the Activated GLP-1 Receptor-G Complex Revealed by Cross-Linking Mass Spectrometry and Integrative Structure Modeling. *ACS Cent Sci* **9**, 992–1007 (2023).
30. E. F. Pettersen, *et al.*, UCSF ChimeraX: Structure visualization for researchers, educators, and developers. *Protein Sci* **30**, 70–82 (2021).
31. R. A. Laskowski, J. Jabłońska, L. Pravda, R. S. Vařeková, J. M. Thornton, PDBsum: Structural summaries of PDB entries. *Protein Sci* **27**, 129–134 (2018).
32. F. Sievers, D. G. Higgins, The Clustal Omega Multiple Alignment Package. *Methods Mol Biol* **2231**, 3–16 (2021).
33. X. Robert, P. Gouet, Deciphering key features in protein structures with the new ENDscript server. *Nucleic Acids Res* **42**, W320–4 (2014).
34. T. J. Dolinsky, *et al.*, PDB2PQR: expanding and upgrading automated preparation of biomolecular structures for molecular simulations. *Nucleic Acids Res* **35**, W522–5 (2007).
35. N. A. Baker, D. Sept, S. Joseph, M. J. Holst, J. A. McCammon, Electrostatics of nanosystems: application to microtubules and the ribosome. *Proc Natl Acad Sci U S A* **98**, 10037–10041 (2001).
36. A. Vangone, R. Spinelli, V. Scarano, L. Cavallo, R. Oliva, COCOMAPS: a web application to analyze and visualize contacts at the interface of biomolecular complexes. *Bioinformatics* **27**, 2915–2916 (2011).
37. A. D. Koromyslova, A. O. Chugunov, R. G. Efremov, Deciphering fine molecular details of proteins' structure and function with a Protein Surface Topography (PST) method. *J Chem Inf Model* **54**, 1189–1199 (2014).
38. Galaxy Community, The Galaxy platform for accessible, reproducible and collaborative biomedical analyses: 2022 update. *Nucleic Acids Res* **50**, W345–W351 (2022). Erratum in: *Nucleic Acids Res* **50**, 8999 (2022).
